# Metabarcoding targeting the EF1 alpha region to assess *Fusarium* diversity on cereals

**DOI:** 10.1371/journal.pone.0207988

**Published:** 2019-01-11

**Authors:** Anne-Laure Boutigny, Angélique Gautier, Ryan Basler, Florian Dauthieux, Stephen Leite, Romain Valade, Jaime Aguayo, Renaud Ioos, Valérie Laval

**Affiliations:** 1 ANSES Laboratoire de la santé des végétaux, Unité de Mycologie, Malzéville, France; 2 INRA, UMR1290 BIOGER_CPP, Thiverval-Grignon, France; 3 ARVALIS Institut du végétal, Thiverval-Grignon, France; Oklahoma State University, UNITED STATES

## Abstract

Fusarium head blight (FHB) is a major cereal disease caused by a complex of *Fusarium* species. These species vary in importance depending on climatic conditions, agronomic factors or host genotype. In addition, *Fusarium* species can release toxic secondary metabolites. These mycotoxins constitute a significant food safety concern as they have health implications in both humans and animals. The *Fusarium* species involved in FHB differ in their pathogenicity, ability to produce mycotoxins, and fungicide sensitivity. Accurate and exhaustive identification of *Fusarium* species *in planta* is therefore of great importance. In this study, using a new set of primers targeting the *EF1α* gene, the diversity of *Fusarium* species on cereals was evaluated using Illumina high-throughput sequencing. The PCR amplification parameters and bioinformatic pipeline were optimized with mock and artificially infected grain communities and further tested on 65 field samples. *Fusarium* species were retrieved from mock communities and good reproducibility between different runs or PCR cycle numbers was be observed. The method enabled the detection of as few as one single *Fusarium*-infected grain in 10,000. Up to 17 different *Fusarium* species were detected in field samples of barley, durum and soft wheat harvested in France. This new set of primers enables the assessment of *Fusarium* diversity by high-throughput sequencing on cereal samples. It provides a more exhaustive picture of the *Fusarium* community than the currently used techniques based on isolation or species-specific PCR detection. This new experimental approach may be used to show changes in the composition of the *Fusarium* complex or to detect the emergence of new *Fusarium* species as far as the *EF1α* sequence of these species show a sufficient amount of polymorphism in the portion of sequence analyzed. Information on the distribution and prevalence of the different *Fusarium* species in a given geographical area, and in response to various environmental factors, is of great interest for managing the disease and predicting mycotoxin contamination risks.

## Introduction

Fusarium head blight (FHB) is a major cereal disease causing severe damage to crops worldwide including Europe. In addition to reducing grain yield and quality, the harvested grains are often contaminated with mycotoxins that are a major health and food safety concern due to their toxicity to humans and other animals. Up to 17 fungal species have been associated with FHB [1], belonging either to the toxinogenic genus *Fusarium* or to the non-toxinogenic genus *Microdochium*. For example, in France, the most common species associated with FHB on soft wheat, durum wheat and barley are *F*. *graminearum sensu lato*, *F*. *avenaceum*, *F*. *poae* and *M*. *nivale*, but species such as *F*. *tricinctum* and *F*. *culmorum* have also been found frequently, along with less frequent species such as *F*. *equiseti*, *F*. *acuminatum*, *F*. *sambucinum*, *F*. *sporotrichioides*, *F*. *verticillioides*, *F*. *heterosporum*, *F*. *subglutinans* and *F*. *oxysporum* [2, 3]. These *Fusarium* species are frequently associated with one another in the infected grains and constitute a disease complex [4]. A similar species profile was also found in other European countries, with local and temporal specificities due to several factors such as climatic conditions, agronomic factors or host genotype [5–12].

Most *Fusarium* species are able to produce one or more mycotoxins with varying degrees of toxicity [5]. *Fusarium* species involved in FHB differ in their epidemiology, pathogenicity, ability to produce mycotoxins, and fungicide sensitivity. However, in a context of strong political and public awareness on the issues of food, health and environmental safety, the current trend is to reduce the quantities of fungicides used on crops, while maintaining efficient and sustainable agriculture. Climate change and evolving cultural farming practices may also contribute to the modification of *Fusarium* species distribution and in turn, mycotoxin content. Information on the distribution and predominance of the different *Fusarium* species in a given geographical area, and in response to various environmental and cultural factors, is therefore of great importance for managing the disease and evaluating mycotoxin contamination risks.

The analysis of *Fusarium* species composition in field samples is mainly performed using morphological identification or species-specific PCR-based methods [2, 13–15]. Morphological identification of *Fusarium* species is based on the microscopic observation of various criteria (mycelia, growth, macroconidia, microconidia, etc.), and requires time and high taxonomic skills. In addition, competition between species during the isolation step can lead to an underestimation of the occurrence of slower-growing species and of morphologically indistinguishable (i.e. cryptic) species, like the *Fusarium graminearum* species complex (FGSC) occurring on cereal spikes and grains. Species-specific PCR-based methods are reliable methods to detect/quantify various *Fusarium* species in field samples [13–15], but constitute *a priori* approaches restricted to known and specific targets. Likewise, target-specific PCR tests cannot be used to search for emerging, i.e. ill-described or yet undescribed, *Fusarium* species.

Typically, identification of fungal species can be achieved by analysis of one or a combination of DNA sequences [16], but still requires preliminary isolation of the fungus in pure culture before amplification by PCR and sequencing of the sequence. The development of more efficient and more affordable high-throughput sequencing techniques now enables the generation of thousands of DNA sequences from environmental samples, without any isolation and culturing step required. Metabarcoding, i.e. the simultaneous amplification and sequencing of barcode sequences directly from environmental samples, has become a very popular and powerful technique to explore the diversity of microorganisms, including slow-competing or uncultivable taxa. Numerous studies have recently described or re-assessed fungal diversity in samples as diverse as soil, rhizosphere, phyllosphere or field samples using metabarcoding [17–24]. High-throughput sequencing methods are evolving at a fast pace, and Illumina MiSeq sequencing is currently the most successful and extensively used technology globally due to its low error rate and the lowest cost per million bases, with a high number of reads generated. It also has a major advantage over other technologies by generating the longer DNA sequences required for barcoding.

For fungi, the internal transcribed spacer (ITS) region of rDNA has been recognized as the official barcode [16]. ITS sequences are largely represented in international databases [16, 25] and have often been used in fungal diversity studies [17, 26]. However, this barcode does not always enable species discrimination for some genera, in particular within *Fusarium* [27]. The translation elongation factor 1-α (*EF1α*) gene, which is involved in the protein translation machinery of eukaryotic cells, has proven to be efficient to distinguish related *Fusarium* species [28, 29], and was selected as the barcode of choice for the FUSARIUM-ID online identification tool [27]. Owing to its phylogenetic utility and the fact that it is present as a single copy in *Fusarium*, this gene appears to be a good candidate for *Fusarium* metabarcoding approaches.

In this study, two PCR primers were designed to amplify an informative region of the *EF1α* gene in the genus *Fusarium* and a bioinformatic pipeline was developed to assess overall *Fusarium* diversity in field samples by metabarcoding. Various control samples were used to validate the precision, sensitivity and robustness of the technique. As a proof of principle, analyses were performed on field samples and the results were compared with real-time PCR species-specific detection tests.

## Materials and methods

### Biological samples

#### Mock communities

Five different fungal mock communities were prepared (Table 1) to test the efficacy of the analytical process (i.e. PCR primer specificity, PCR reaction, bioinformatic pipeline) in differentiating *Fusarium* species. Mock communities 1 to 4 included various *Fusarium* species and other fungal plant pathogens from diverse taxonomic groups from the Anses, ARVALIS-Institut du végétal and INRA laboratory collections. All of the strains used in this publication were single-spored. They were characterised by morphological identification when possible and by sequencing of at least two genes: *ITS* and *EF1α* (S1 Fig). The sequences of *ITS*, *EF1α* and some of *RNA polymerase II* second largest subunit *(RPB2)* are publicly available on R-Syst::fungi database (http://138.102.89.206/new_rsyst_chp/, https://github.com/r-syst/databases/tree/master/r-syst::fungi). To search for these sequences enter internal lab id (available S1 Table column 2) in ‘advance search’ ‘strain’ and then click to view the results. Using Phylogeny.fr [30] and the following set up: MUSCLE alignment, Gblock curation, Maximum Likelihood PhyML, a phylogenic tree was built with *EF1α* sequences of our strains and *EF1α* sequences from collection strains of the Fusarium ID database [27] (S2 Fig). To confirm the name, the same way an *RPB2* phylogenic tree was obtained of some of our strains (S3 Fig). Mock community 5 contained 10 species in the FGSC previously obtained from the ARS culture collection (USDA ARS, Peoria, Illinois, USA). To prepare each mock community, 100 ng of DNA of each species were mixed in sterile water, except for mock community 5 for which 10 ng of DNA of each species were mixed.

**Table 1:** DNA mock communities description.

Mock 1		Mock 2		Mock 3	
Species	ID	Species	ID	Species	ID
***Fusarium oxysporum***	FOR 4	***Fusarium equiseti (****FIESC 14)*	C33	***Fusarium culmorum***	MTK0055
***Fusarium sambucinum***	C39	***Fusarium graminearum***	MTK0019	***Fusarium poae***	MTK0005
***Fusarium sp*. *(FSSC 11)***	FJMO	***Fusarium sambucinum***	C39	***Fusarium pseudonygamai***	FoxFMO14
***Fusarium* sp.**	22374	*Cladosporium delicatulum*	C58	***Fusarium sambucinum***	C39
***Fusarium staphyleae***	22316	*Cladosporium sphaerospermum*	96	***Fusarium sporotrichioides***	Fs23
*Alternaria alternata*	C61	*Colletotrichum orbiculare*	*C-orbiculare*	*Calonectria pseudonaviculata*	93
*Botrytis cinerea*	C87	*Eutypa lata*	0002–11	*Epicoccum nigrum*	100
*Helminthosporium tritici-repentis*	MTK0038	*Gaeumannomyces graminis var*. *tritici*	MTK0077	*Leptosphaeria maculans*	102
*Phaeoacremonium mortoniae*	0002–12	*Microdochium nivale*	Mn08N58	*Microdochium majus*	MTK0025
*Puccinia triticina*	B77Saba	*Pseudocercosporella herpotrichoides*	MTK0049	*Mortierella elongata*	95
*Parastagonospora nodorum*	MTK0081			*Pseudonectria buxi*	94
*Sclerotinia sclerotiorum*	C62				
Mock 4		Mock 5			
Species	ID	Species	ID		
***Fusarium culmorum***	MTK0009	***Fusarium cortaderiae***	NRRL31171		
***Fusarium langsethiae***	Fl39	***Fusarium meridionale***	NRRL29010		
***Fusarium sambucinum***	C39	***Fusarium acaciae-mearnsii***	NRRL26755		
***Fusarium subglutinans***	Fsub69	***Fusarium austroamericanum***	NRRL28718		
***Fusarium temperatum***	Fpro32-2	***Fusarium aethiopicum***	NRRL46722		
***Fusarium sp*. *(FTSC)***	FT12	***Fusarium brasilicum***	NRRL31238		
***Fusarium avenaceum***	MTK0070	***Fusarium vorosii***	NRRL38208		
***Fusarium verticillioides***	Fmoni62	***Fusarium gerlachii***	NRRL38380		
*Leptosphaeria biglobosa*	C68	***Fusarium mesoamericanum***	NRRL25797		
*Microdochium bolleyi*	98	***Fusarium asiaticum***	NRRL13818		
*Zymoseptoria tritici*	MTK0030				

This table presents the species name and identification number (ID) of the strains used to prepare the 5 mock communities. DNA of these strains was extracted and mixed in equimolar quantity after Qubit quantification.

#### Artificially infected grains

Infected grain communities were prepared (Table 2) to (i) test the efficacy of the analytical process to differentiate *Fusarium* species in complex samples, (ii) verify consistency between replicates and DNA extractions, and (iii) evaluate the sensitivity of the technique. Healthy wheat grains were infected *in vitro* with *Fusarium* strains from the ANSES, ARVALIS and INRA laboratory collections. Each *Fusarium* strain was grown on potato dextrose agar (PDA) plates for 7 to 10 days. Wheat grains were sterilized by autoclaving, and plated on PDA. For each *Fusarium* strain, a series of plates containing 6 to 8 wheat grains was prepared. Three plugs taken from the edge of the fungal cultures on PDA were evenly placed on each plate containing the grains, and incubated for 10 days to allow fungal colonization of the grains. After infection, grains were dried for 24 h at 50°C.

**Table 2:** Description of artificially infected grain samples prepared in this study.

Sample name	Number of artificially infected grains									Number of uninfected grains
	*F*. *graminearum*	*F*. *langsethiae*	*F*. *poae*	*F*. *sp*. *FTSC*	*F*. *culmorum*	*F*. *avenaceum*	*F*. *subglutinans*	*F*. *verticillioides*		
G10 P1 L1 T1	10	1	1	1					87	
G10 L1 P1	10	1	1						88	
G10 L1 T1	10	1		1					88	
G10 P1 T1	10		1	1					88	
F8 100	1	1	1	1	1	1	1	1	92	
F8 200	1	1	1	1	1	1	1	1	192	
F8 500	1	1	1	1	1	1	1	1	492	
F8 1000	1	1	1	1	1	1	1	1	992	
F8 2000	1	1	1	1	1	1	1	1	1992	
F8 5000	1	1	1	1	1	1	1	1	4992	
F8 10000	1	1	1	1	1	1	1	1	9992	

This table describe which *Fusarium* species were used to inoculate grain and the number of infected grains and uninfected grains per samples.

A first set of 100 grain samples containing 10 grains infected with *F*. *graminearum* (LSV M808) and 1 grain infected with *F*. *langsethiae* (LSV M846), *F*. *poae* (LSV M861) and *F*. *tricinctum* (LSV M860) in different proportions was prepared (Table 2). Each sample was prepared with durum wheat (cultivar CG09) and soft wheat (cultivar Galopain). The samples in their entirety were ground in a grinding jar using a TissueLyser (Qiagen, France). The resulting powder (particle size not determined) was collected in a 50 mL falcon tube and mixed manually before collecting 200mg for DNA extraction.

A second set of artificially infected samples was prepared by mixing 8 grains individually infected with *F*. *poae* (FP1), *F*. *langsethiae* (FL33), *F*. *sp*. *FTSC* (FT12), *F*. *culmorum* (C17), *F*. *avenaceum* (Fa), *F*. *graminearum* (FG20), *F*. *subglutinans* (FU12238) and *F*. *verticillioides* (FU09041), respectively, with a total of 100, 200, 500, 1000, 2000, 5000 and 10,000 healthy grains (Table 2). Each sample was prepared with durum wheat (cultivar Miradou). The samples in their entirety were ground using a PULVERISETTE 14 Variable-Speed Rotor Mill (Fritsch, Germany). The particle size of the flour was approximately 5μm.

#### Field samples

In 2014 and 2015, 65 field samples of durum wheat, soft wheat and barley were collected in different locations in France. Samples were selected to represent a variety of cereal-growing regions with different climates and cultural farming practices, and following different chemical treatment strategies (S4 Fig). The samples were used to test the applicability of the protocol and analysis steps. No permits were required for the field samplings and the authors have permission to conduct the study. Each sample, corresponding to 3 kg of grains, was homogenised and ground using a hammer mill (TITAN 2000, fao). Then, 100 g of flour was ground again using an MM400 Retsch mixer mill (Retsch, Germany). The particle size of the flour was approximately 5**μ**m.

### Methods

#### DNA extraction

For the mock communities, fungal DNA was obtained from aerial mycelium aseptically scraped from cultures grown on either PDA or malt agar. The mycelium was lyophilized for 24h in an Edwards Modulyo 4K Lyophilizer (Edwards, United Kingdom) and 100 mg of lyophilized mycelium was placed in a Fast-Prep tube (2 mL) containing 130 mg of glass beads (4.5 mm in diameter; Dutscher, France) and ground using FastPrep (MP Biomedicals, France) for 30s at maximum shaking frequency. DNA was extracted using a DNeasy Plant Mini kit (Qiagen, France) according to the manufacturer’s instructions, except that the incubation time was extended to 1 h at 65°C, and the volumes of AP1 and P3 buffers were doubled.

DNA was extracted in the same way from both artificially infected grains and field grain samples. Three independent extractions were performed per sample for artificially infected samples with four species (Table 2), whereas only a single extraction was carried out for artificially infected samples with eight species (F8 100 to F8 10,000; Table 2) and field samples. 100 mg of the homogenized flour was re-suspended in 400 μL of AP1buffer (DNeasy Plant Mini kit, Qiagen, France) and sonicated for 30s. Then, 10 μL of proteinase K (10 mg/mL) and 10 μL of RNase A (Qiagen) were added to the suspension. After 20 minutes of incubation at 65°C, the protocol recommended by the manufacturer was followed. All DNA extracts were quantified using a Qubit 2.0 Fluorometer (Invitrogen, USA).

#### Primer design

A local database of partial *EF1α* gene sequences from different *Fusarium* species was constructed, containing sequences downloaded from the Fusarium ID database [27]. In total, the database contained 162 sequences, covering 74 species. Using Phylogeny.fr [30], a phylogenic tree was built and 6 divergent sequences were selected and aligned using MultAlin [31] with *EF1α* sequences from other genera such as *Zymoseptoria*, *Puccinia*, *Microdochium*, *Parastagonospora*, and *Leptosphaeria* in order to find regions i) sufficiently conserved in *Fusarium* to design a genus-specific primer pair, and ii) flanking a DNA region showing a high level of interspecific polymorphism within the genus *Fusarium*. Several sets of candidate primers were designed and their specificity was first assessed *in silico* by BLAST with the NCBI database, then *in vitro* by PCR using DNA from a panel of 54 strains including 31 *Fusarium* species and 19 other genera of fungi (S5 Fig). The best primer pair was then selected: EF1-F2 (5’TCATCGGCCACGTCGACTCT3’) and EF1-R3 (5’TACCAGCCTCGAACTCACCA3’) (S6 Fig).

#### PCR and Illumina sequencing

In order to obtain a PCR fragment suitable for Illumina MiSeq sequencing, primers EF1-F2 and EF1-R3 were tagged with specific sequences (PYROFUS-EF1-F2-MISEQ: 5’**CTTTCCCTACACGACGCTCTTCCGATCTATTAACCCTCACTAAAGGGA**TCATCGGCCACGTCGACTCT3’ and PYROFUS-EF1-R3-MISEQ 5’**GGAGTTCAGACGTGTGCTCTTCCGATCTCCCTATAGTGAGTCGTATTA**TACCAGCCTCGAACTCACCA3’). The PCR reaction contained 1 x mastermix for the Type-it Microsatellite PCR Kit (Qiagen, France), 5 μl Q-Solution and 0.25 μM of each primer in a total volume of 50 μL. PCR reactions were performed with either 60 ng (mock communities) or 125 ng (artificially infected grains and field samples) of DNA. PCR reactions were performed using an Applied Biosystems 9700 thermocycler. The amplification parameters consisted of an initial denaturation step at 95°C for 5 min; then either 31 or 35 cycles of denaturation at 95°C for 60 s, annealing at 65°C for 90 s, and elongation at 72°C for 60 s; and a final extension step at 72°C for 10 min. The size of the final PCR amplicon before sequencing was around 750 bp. For field samples, in order to obtain a sufficient amount of PCR product for Illumina MiSeq sequencing, 3 or 6 independent PCRs of the same sample where pooled before purification. The PCR products were purified by PEG-8000 precipitation and concentration (26.2% polyethylene glycol 8000, 6.6 mM MgCl_2_ and 0.6 M NaOAc) and sent to the GenoToul platform (INRA Toulouse) for Illumina MiSeq (V3 2 x 250 bp) sequencing. Three independent sequencing runs were performed.

#### Species detection by real-time PCR

Total DNA from each of the 65 field samples was analyzed by species-specific real-time PCR targeting *F*. *avenaceum*, *F*. *culmorum*, *F*. *graminearum*, *F*. *langsethiae*, *F*. *poae*, *F*. *sporotrichioides* and *F*. *tricinctum* [32]. The artificially infected samples were also analyzed with these tests in order to verify the presence of the *Fusarium* species inoculated. The primers and probes used were custom-synthesized by Eurogentec (Seraing, Belgium) and are listed in Table 3.

**Table 3:** Primer and probe sequences and real-time PCR amplification conditions for each *Fusarium* species (table extracted from [32]).

Target species	Name	Sequence *	Final concentration	Annealing temperature
***F*. *graminearum***	EF1-FCFG_F	TCGATACGCGCCTGTTACC	300nM	62°C
	EF1-FG_R	ATGAGCGCCCAGGGAATG	300nM	
	grami2-EF1_rev	AGCCCCACCGGGAAAAAAATTACGACA	100nM	
***F*. *culmorum***	EF1-FC_F2	CGAATCGCCCTCACACG	300nM	62°C
	EF1-FC-R2	GTGATGGTGCGCGCCTAG	300nM	
	culmo2-EF1-R2	ATGAGCCCCACCAGAAAAATTACGACAA	100nM	
***F*. *poae***	EF1-FP2_F	CTCGAGCGATTGCATTTCTTT	300nM	60°C
	EF1_FP2_R	GGCTTCCTATTGACAGGTGGTT	300nM	
	EF1-FP	CGCGAATCGTCACGTGTCAATCAGTT	100nM	
***F*. *sporotrichioides***	EF1-FS_F3	GGCTCATACCCCGCCG	300nM	60°C
	EF1-FS_R2	GCGCCCATGTAAATGGATG	300nM	
	EF1 FS	TGGGAAGGGCAAAAGCGCCTGT	100nM	
***F*. *langsethiae***	EF1 FL F3	GCCGTGTCGTAATTTTTTTTGTG	300nM	62°C
	EF1 FL R3	AAATGGCTATGTGGGAAGGAAG	300nM	
	EF1_FL	GGGCTCATACCCCGCCACTCGA	300nM	
***F*. *avenaceum***	EF1-FA_F2	CATCTTGCTAACTCTTGACAGACCG	300nM	64°C
	EF1-FA_R3	GGGTAATGAATGCGTTTCGAA	300nM	
	Ef1 FA	AGCGAGTCGTGGGAATCGATGGG	150nM	
***F*. *tricinctum***	EF1-FT_F2	CCACGATTCGCTCCCTCAC	300nM	62°C
	EF1-FT_R2	GGTAAGATACCCCACCAGAAAAA	300nM	
	EF1 FT	AGCGGGGTAATGGATGCGTTTCGAGT	150nM	

*All probes were labelled with FAM fluorophore and BHQ1 Quencher

Real-time PCR assays were performed using 2 x qPCR MasterMix with ROX and Uracil N Glycosylase (UNG) from Eurogentec (Angers, France). PCR reactions were performed in a total of 20 μL consisting of 12.5 μL of mastermix, with primers and probes at the concentrations described in Table 3, and 5 μL of DNA extract (100 ng for field samples) were added to the mix. Standard calibration curves were constructed with DNA from a pure culture of the target species with concentrations ranging from 1 ng/μL to 0.001 ng/μL and were included in each experiment. Real-time PCR reactions were performed on an ABI PRISM 7900 Sequence Detection System (Applied Biosystems, Foster City, CA, USA) in Applied Biosystem 96-well plates. Cycling conditions included an initial denaturation step at 95°C for 10 min, followed by 40 cycles each at 95°C for 15 s then one minute in specific hybridization/polymerization conditions (Table 3). Each DNA template was analyzed in triplicate.

#### Filtering, processing and assigning a taxonomic classification to sequences

A stringent pipeline taking as a basis for the MiSeq SOP [33] was used to filter and process raw sequences utilizing MOTHUR v.1.36 [34] (S7 Fig). After FastQC analysis [35], the reverse sequences were associated with poor quality files. Around 100 bp of each read received quality scores lower than 20. We eliminated these poor-quality reads from further analyses. By contrast, forward sequences met good quality standards and were processed. First, only the sequences showing the expected primer and barcode sequences were retained. Then, sequences with low quality (shorter than 100 bp or including low quality scores) were removed. After preprocessing the sequences, multiple sequence files were merged and unique sequences represented by singletons removed. Chimeric sequences were removed with the Uchime tool [36] available in MOTHUR, using self as the reference. Uncorrected pairwise distances were determined between the remaining sequences with the Needleman alignment method [37] with match 1.0, mismatch -1.0, gapopen -2.0 and gapextend 1.0 using 1000 iterations. The distance matrix was then clustered with the average neighbor algorithm to assign sequences to operational taxonomic units (OTUs).

A custom *EF1α* gene fungal reference database with 9,196 sequences was created by extracting Fungal *EF1α* sequences from GenBank using the query: (fungi[All Fields] OR fungi[Organism] OR fusarium[Title]) AND (translation elongation factor[Title] OR tef1[Title] OR translation elongation factor-1 alpha[Title] OR elongation[Title] OR EF1-a[Title] OR tef-1[Title]) AND (CBS*[Title] OR NRRL*[Title] OR MUCL*[Title] OR voucher*[Title] OR DAOM*[Title] OR MAFF*[Title] OR MIAE*[Title] OR IPO*[Title] OR ATCC*[Title] OR LMSA*[Title]) AND (100[SLEN]: 3000[SLEN]) NOT Fusarium sp.[Title] NOT IonTorrent[All Fields] NOT (TEF3[Title] OR translation elongation factor 3[Title] OR tef-3[Title] OR elongation factor 3[Title] OR EF-3[Title]). An additional set of partial *EF1α* gene sequences was generated in-house by PCR, using the EF1-F2/EF1-R3 primers and DNA from the *Fusarium* strains available in the INRA and ANSES collections. The custom *EF1α* gene fungal reference database was called ‘merge.reference.fasta’ in the pipeline described in the S7 Fig. There were nine taxonomic levels associated with each fungal sequence in the following order: kingdom, phylum, subphylum, class, subclass, order, family, genus, and species, with undefined levels recorded as unclassified. The classification of taxonomic levels for sequences was manually curated from the Mycobank database [38]. The curated taxonomy file was called ‘merge.taxonomy.fasta’ in the pipeline described in the S7 Fig. The consensus taxonomy for representative sequences for each OTU was performed using MOTHUR’s classifier [39] to assign the taxonomy of sequences from the custom *EF1α* fungal reference database using 2% divergence. An excel database was created containing OTU number, read count per OTU per sample, representative sequence name, representative sequence, and OTU representative assignation. Then, the OTUs were carefully checked and assigned to the species taxonomic level for *Fusarium* OTUs and genus taxonomic level for non-*Fusarium* OTUs. OTUs with the same species or genus assignment were merged and were represented as a percentage of the total sequences in the sample, with a minimum acceptance confidence level. Errors in the sequence of mock communities were calculated using Sanger sequences as references (MOTHUR v.1.36; [34]). The calculated errors were then used to determine the minimum acceptance confidence level to retain OTUs (S7 Fig).

#### Statistical analysis

A linear regression model in R version 3.3 with Rcmdr [40] was used to evaluate the influence of PCR cycle number, run and PCR pooling on the percentage of final *Fusarium* OTUs.

To compare the diversity present in each wheat sample, only *Fusarium* species determined from OTUs as described above were considered, as the experiment was developed specifically for *Fusarium* analysis. Analyses were performed with R (R version 3.3.1 (2016-06-21)) using the ‘Phyloseq’ R package specifically designed for analysis of microbiological communities [41]. Using ‘Phyloseq’, a Heatmap of abundance of each species per sample was generated.

## Results

### Design of a *Fusarium*-specific PCR primer pair

The different PCR primers were evaluated on a panel of DNA extracts representing 30 *Fusarium* species and 23 non-*Fusarium* species. Primers EF1-F2 and EF1-R3 were selected due to their specificity for the *Fusarium* genus (S5 Fig), and their ability to generate a single sharp band after electrophoresis on an agarose gel (data not shown). The primers amplified a 640 bp region of the *EF1α* gene in the *Fusarium* species, displaying a high level of interspecific polymorphism. This region was successfully amplified with DNA from the 30 *Fusarium* species tested. No PCR amplification was observed with DNA from uninfected wheat. Weak amplification signals were observed for a few non-target taxa, such as *Alternaria alternata*, *Botrytis cinerea*, *Sclerotinia sclerotiorum*, *Zymoseptoria tritici*, *Leptosphaeria maculans*, *Pseudocercosporella herpotrichoides*, *Phaeoacremonium mortoniae*, *Epicoccum nigrum*, *Eutypa lata*, *Cladosporium delicatulum*. and *Colletotrichum orbiculare*. (S5 Fig).

### Analysis of the DNA mock communities

The five fungal mock communities were used as positive controls in three independent sequencing experiments. In these three runs, a total of 3,491,565 raw reads were generated for the mock communities. After filtering, 81,604 (2.3% of the raw sequences) were kept for the analysis. Mock communities were used to determine error rates. As the *EF1α* Sanger sequence of each strain included in each mock community was available, a maximum error rate generated by Illumina MiSeq sequencing was calculated for each mock community using Mothur (S7 Fig). In our experiments, the maximum error rate obtained was 0.3%. In this respect, for each sample analyzed (artificially infected grain samples and field samples), OTUs representing less than 0.3% of the total reads were removed from the data.

**Fig 1:**
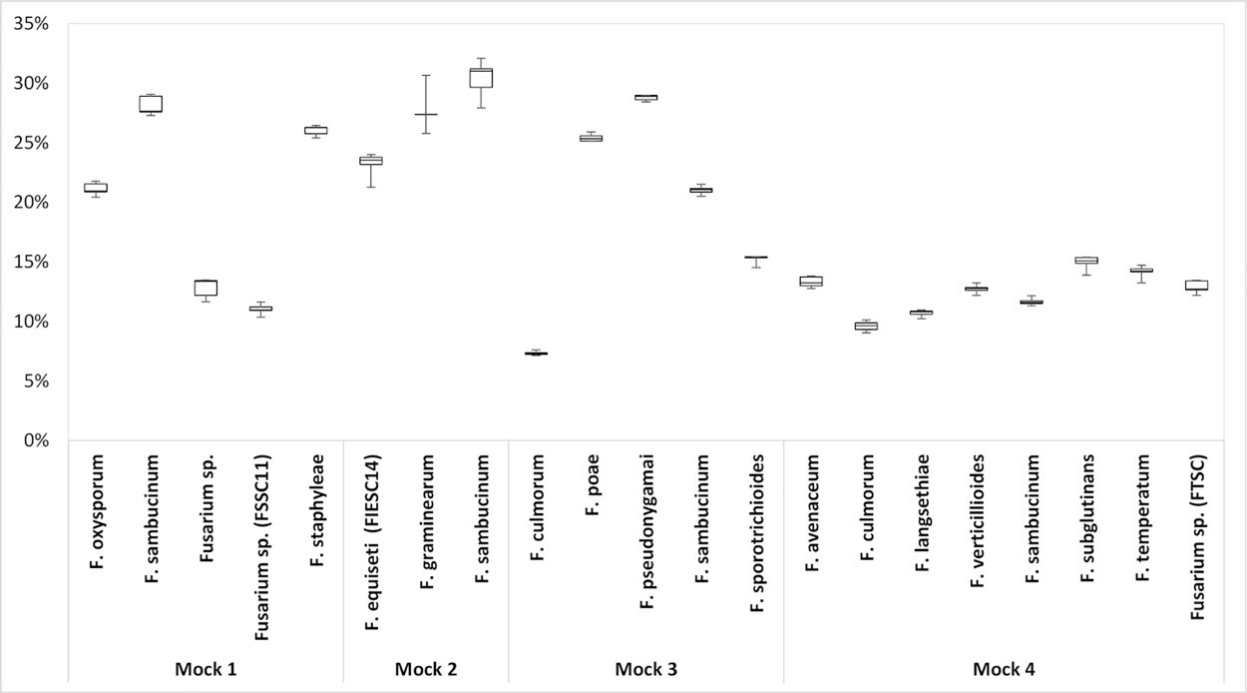
Boxplots of relative abundance of reads per species for mock communities 1 to 4. For each species, each Boxplot includes 8 independent samples for each mock (two PCR cycle conditions and 2 replicates for sequencing run 1, one PCR cycle condition and 3 replicates for sequencing run 2, one PCR cycle condition and 1 replicate for sequencing run 3).

For each OTU, results were expressed as a percentage of sequences out of the total number of sequences analyzed per sample. The expected *EF1α* sequences from all the *Fusarium* species added in mock 1, mock 2, mock 3 and mock 4 could be identified (Fig 1). The percentage of *Fusarium* OTUs did not differ significantly (*p* > 0.05) between the different PCR cycle conditions tested (31 or 35 cycles), and between runs (3 independent runs). Two independent PCRs were also performed with DNA from the same mock community and sequenced in one run, and no difference could be observed between the percentages of *Fusarium* OTUs retrieved. Sequences from a few non-*Fusarium* species that were included in the mock communities were identified: *Phaeoacremonium* (mean 1.0%, SEM 0.1%) for mock 1, *Cladosporium* (mean 7.3%, SEM 2.9%), *Colletotrichum* (mean 10.7%, SEM 2.8%), *Eutypa* (mean 0.4%, SEM 0.3%), and *Pseudocercosporella* (mean 0.4%, SEM 0.1%) for mock 2, and *Leptosphaeria* (mean 2.2%, SEM 0.2%) for mock 3, *Zymospetoria* (mean 0.2%, SEM 0.002%) for mock 4. No other species/genera were identified.

In mock community 5, which contained 10 species from the FGSC, only three species could be identified based on the sequence analyses: *F*. *acaciae-mearnsii* (mean 5.3%, SEM 0.1%), *F*. *cortaderiae* (mean 10.9%, SEM 0.1%), and *F*. *meridionale* (mean 5.6%, SEM 0.1%). All the other reads were assigned to *F*. *graminearum* (77.8%, SEM 0.1%). This correlates with the differences observed in the 200 bp sequence of the *EF1α* gene originating from Illumina Miseq sequencing, as *F*. *cortaderiae*, *F*. *meridionale* and *F*. *acaciae-mearnsii* have 4, 3 and 3 base pair differences, respectively. The other species of the FGSC showed fewer than 3 base pair differences in the 200 bp sequence of the *EF1α* gene used for the analysis, and could not be differentiated using the 2% sequence divergence threshold.

### Analysis of the artificially infected grain samples

A total of 6,995,829 raw sequences were generated for the infected grain communities, and after filtering, 172,894 unique sequences were kept (2.4% of the raw sequences).

**Fig 2:**
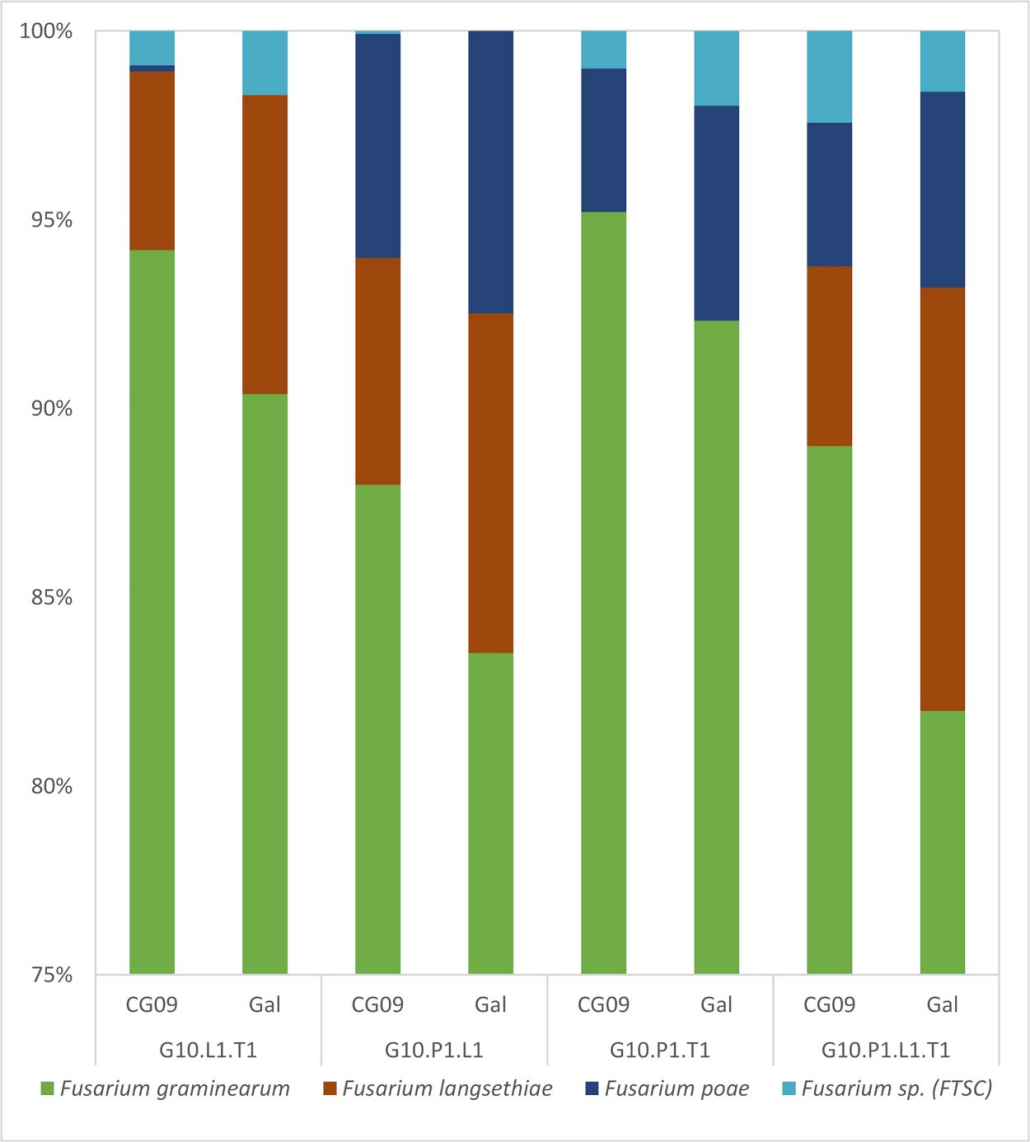
Percentage of sequences in Fusarium OTUs retrieved from artificially infected grain samples. G10 = 10 grains infected with *F*. *graminearum*; L1 = 1 grain infected with *F*. *langsethiae*; P1 = 1 grain infected with *F*. *poae*; T1 = 1 grain infected with *F*. *tricinctum*; CG09 and GAL = wheat varieties. The scale was adjusted as *F*. *graminearum* accounted for more than 75% of the reads for each sample.

In samples contaminated with 4 species, the results showed that all these species could be retrieved in the mixed samples, regardless of the level and type of artificial contamination, the wheat varieties or the DNA extraction replicate (Fig 2). The results obtained comparing these 24 DNA samples (4 types of artificial contamination, 2 varieties, 3 extractions), sequenced in one run, were reproducible: *F*. *graminearum* (mean 89.4%, SEM 4.5%), *F*. *langsethiae* (mean 7.2%, SEM 2.5%), *F*. *poae* (mean 5%, SEM 1.6%), and *F*. *tricinctum* (mean 1.6%, SEM 0.5%). *F*. *graminearum* represented the highest percentage of reads as 10 grains infected with *F*. *graminearum* were mixed with only one single grain infected with the other species. *EF1α* sequences of *F*. *poae*, *F*. *tricinctum* or *F*. *langsethiae* could be detected in samples with only one single grain infected, mixed with 100 healthy grains. Real-time PCR analysis confirmed the presence of the 4 different *Fusarium* species in the samples (data not shown).

**Fig 3:**
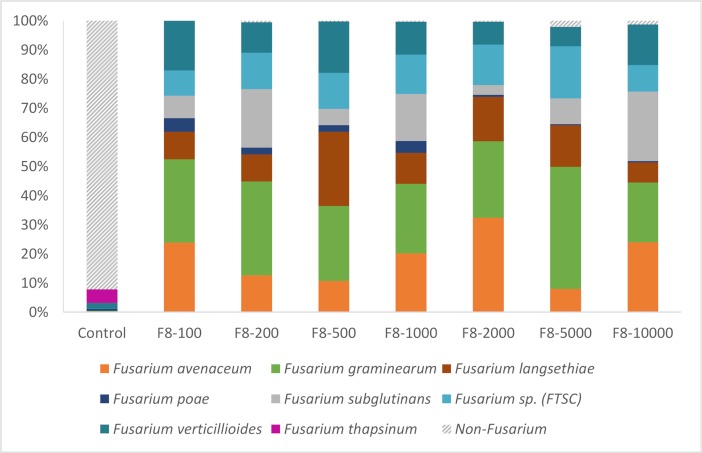
Percentage of sequences in *Fusarium* OTUs retrieved from artificially-infected grain samples. Eight grains infected with *F*. *poae*, *F*. *langsethiae*, *Fusarium sp*. *(FTSC)*, *F*. *culmorum*, *F*. *avenaceum*, *F*. *graminearum*, *F*. *subglutinans* and *F*. *verticillioides* were added to increased the number of uninfected grains. The same lot of uninfected grains was used as the control sample.

As the results were reproducible regardless of the wheat variety, it was decided to use only one variety to assess the sensitivity of the technique (F8-100 to F8-10,000, Table 2). In addition, as no significant effect of extraction and PCR cycle number were observed during the preliminary tests, these samples were extracted once, and tested with PCR cycling condition described in the materials and methods. The products of 6 independent PCRs were pooled per sample to improve sensitivity. *EF1α* sequences of *F*. *avenaceum*, *F*. *graminearum*, *F*. *langsethiae*, *F*. *poae*, *F*. *subglutinans*, *Fusarium sp*. *(FTSC)* and *F*. *verticillioides* were identified in all samples (99.3% ± 0.74% SEM all sequences) (Fig 3). No sequences of *F*. *culmorum* were observed. A two- to four-fold increase in non-*Fusarium* sequences was observed with higher levels of dilutions (F8-5000 and F8-10,000). In the control sample, 38,977 sequences were kept after filtering, of which 92.1% did not correspond to *Fusarium* OTUs. 4.6% of the reads were identified as *Fusarium thapsinum* and 1.9% as *F*. *verticillioides*. *F*. *thapsinum* was not identified in the other samples although the wheat seeds used all originated from the same sample.

### Analysis of field samples

**Fig 4:**
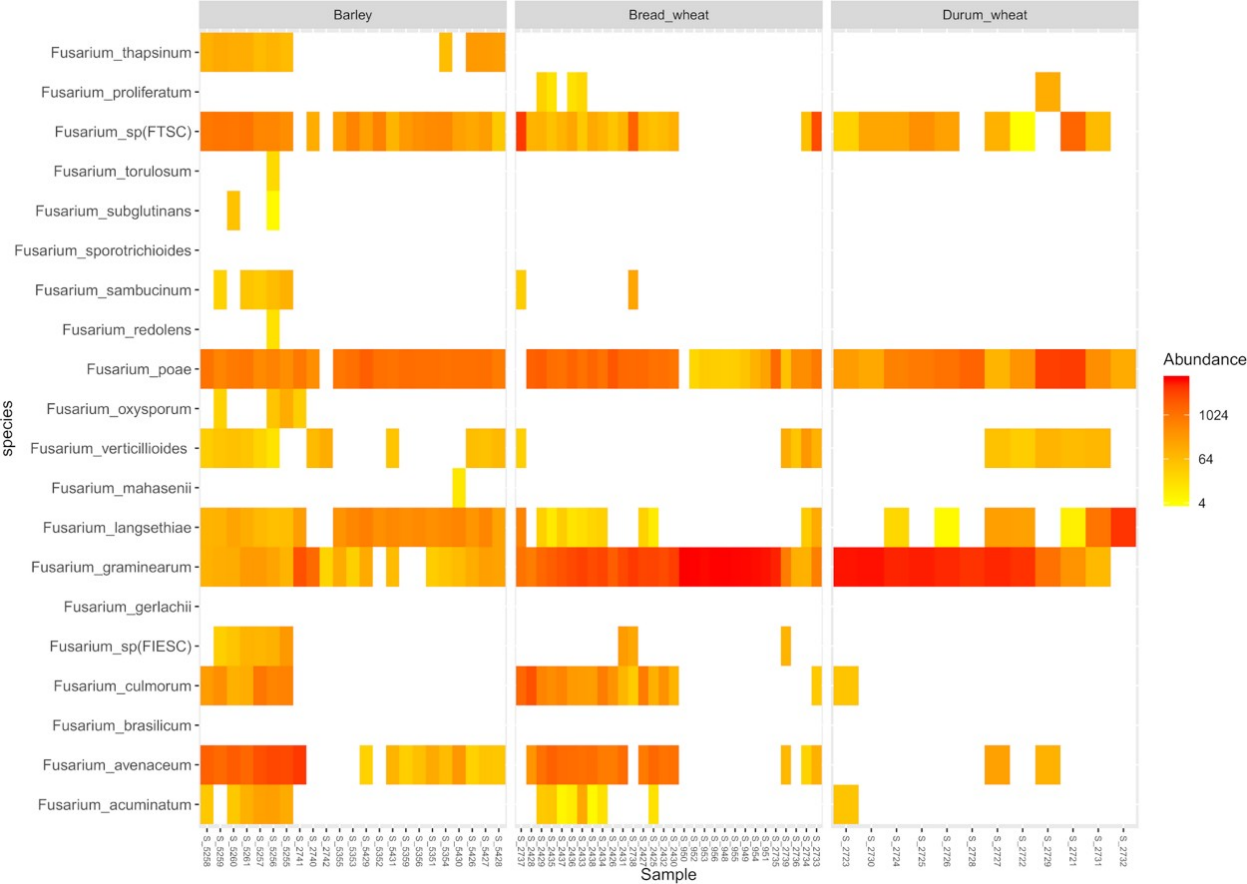
Heatmap analysis of *Fusarium* species recovered from field samples. The colour reflects the read abundance: from fewer reads in yellow to increased number of reads in red.

In all, 65 field samples were analyzed using the protocol developed in this study. Samples were collected from 12 fields of durum wheat, 30 fields of soft wheat, and 23 fields of barley in 2014 and 2015 (S1 Fig). A total of 5,115,242 raw sequences were generated. A total of 141,103 unique sequences remained after filtering (2.8% of the raw sequences). In order to obtain a sufficient amount of PCR amplicons before Illumina sequencing, independent PCR amplifications of the same DNA template were pooled. A first test showed that the percentage of *Fusarium* OTUs or the total percentage of non-*Fusarium* OTUs did not differ significantly (*p* > 0.5) between pooling 3 versus 6 PCR reactions before sequencing. Thus, 6 independent PCRs were performed for all samples and pooled before sequencing as this increased the amount for detection. The Heatmap (Fig 4) shows the abundance of each species detected. The abundance corresponds to the percentage of the number of reads per species out of the total number of reads in a sample. Up to 11 *Fusarium* species could be detected in wheat and up to 16 in barley. *F*. *graminearum* and *F*. *poae* were present in the majority of the samples. *Fusarium sp*. *(FTSC)*, *F*. *culmorum*, *F*. *langsethiae*, *F*. *verticillioides* and *F*. *avenaceum* were also detected in many samples, regardless of the type of cereal. Some *Fusarium* species were only detected in wheat, such as *F*. *proliferatum*, whereas by contrast, other *Fusarium* species, such as *F*. *thapsinum*, *F*. *torulosum*, *F*. *subglutinans*, *F*. *redolens*, *F*. *oxysporum*, and *F*. *mahasenii*, were only observed in barley.

The DNA extracts from the 65 field samples were also analyzed by seven species-specific real-time PCR tests targeting *F*. *graminearum*, *F*. *culmorum*, *F*. *poae*, *F*. *tricinctum*, *F*. *langsethiae*, *F*. *sporotrichioides* and *F*. *avenaceum*, respectively (S1 Table). These real-time PCR tests were developed in 2009 using the *Fusarium* species taxonomy that was in force at that time. Since then, new cryptic species have been described, like *F*. *sibiricum* which is closely related to *F*. *sporotrichioides*, or taxa from the *F*. *graminearum* or *F*. *tricinctum* species complex. These cryptic species will therefore not be differentiated with the real-time PCR tests used here. Comparison of the real-time PCR results and the metabarcoding analyses is summarized in Table 4. Only a few discrepancies were observed. The level of discrepancy varied according to the *Fusarium* species. Similar results were obtained using the two techniques for *F*. *culmorum*, *F*. *poae* and *F*. *sporotrichioides* (< 8% of discrepant results), whereas a higher percentage of discrepant results was observed for *F*. *avenaceum*, *F*. *graminearum*, *F*. *langsethiae* and *F*. *tricinctum (*18.46% to 36.92%). Except for *F*. *tricinctum*, the metabarcoding always detected more positive samples than real-time PCR did.

**Table 4:** Discrepancy analysis results for 65 field cereal samples by real-time PCR and PCR/Illumina sequencing.

Detection	*F*. *avenaceum*	*F*. *culmorum*	*F*. *graminearum*	*F*. *langsethiae*	*F*. *poae*	*F*. *sporotrichioides*	*F*. *tricinctum*
qPCR / metabarcoding							
**+/+**	23	24	49	17	62	0	40
**-/-**	26	40	4	24	1	60	11
**+/-**	2	1	0	1	2	5	8
**-/+**	14	0	12	23	0	0	6
Discrepancies (in % of samples of non-corresponding detection out of total samples)	24.62	1.54	18.46	36.92	3.08	7.69	21.54

This table summarised the number of samples in which *Fusarium* species were detected by qPCR or the metabarcoding techniques developed in this paper. For each species, the number of samples that were positively detected by both qPCR and the metabarcoding technique (+/+), the number samples negatively detected by both techniques (-/-) and the number of samples positively detected only by qPCR (+/-) or only by metabarcoding (-/+) are presented. A percentage of discrepancies in detection is calculated at the bottom of the columns for each species.

**Fig 5:**
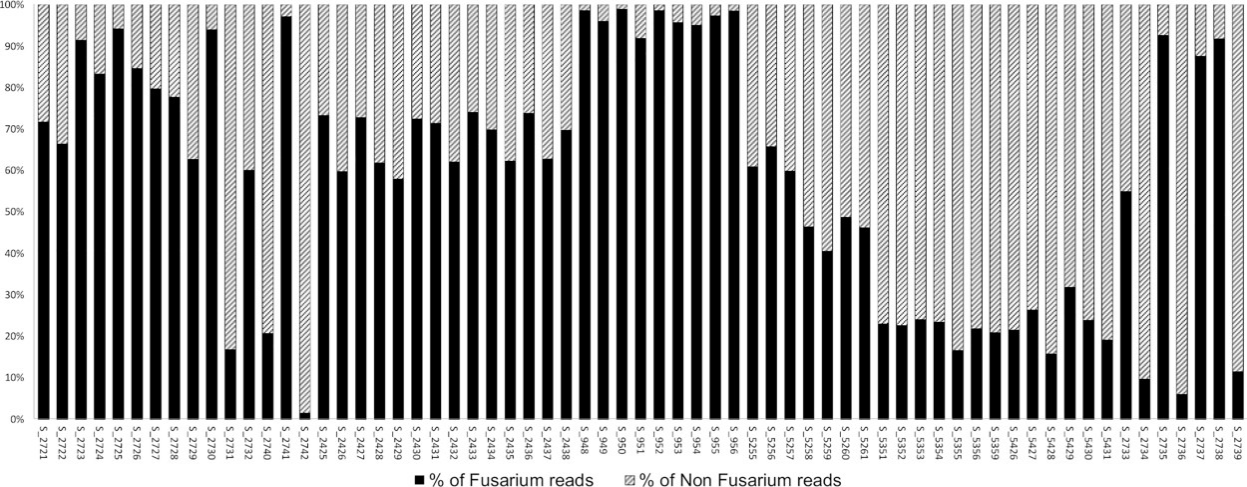
Percentage of *Fusarium* and non-*Fusarium* reads per sample after bioinformatic analyses. Percentage of *Fusarium* reads are presented in black and percentage of non *Fusarium* reads in hatched per field sample.

*EF1α* sequences from other genera were also identified. The percentage of reads for non-*Fusarium* species varied from 5% to 95% of total reads after filtering (Fig 5). The percentage of non-*Fusarium* reads increased when contamination by *Fusarium* species was low. For example, small amounts of *Fusarium* species were detected by real-time PCR in samples S_2742, S_2734 and S_2736, in which up to 95% of non-*Fusarium* species were identified by metabarcoding. The non-*Fusarium* taxa identified in durum wheat were *Alternaria*, *Cladosporium*, *Colletotrichum*, *Ilyonectria*, and *Lewia*; whereas *Alternaria*, *Campylocarpon*, *Chalastospora*, *Cladosporium*, *Colletotrichum*, *Diaporthe*, *Lewia*, *Trichoderma* and *Zymoseptoria* were detected in bread wheat and *Alternaria*, *Cladosporium*, *Colletotrichum*, *Gnomoniopsis*, *Lewia*, *Trichoderma* and *Zymoseptoria* were detected in barley.

## Discussion

This study presents the development and validation of a metabarcoding-based protocol to assess *Fusarium* diversity on cereals. The protocol includes a method for extracting good quality DNA, followed by PCR amplification of a genus-specific, polymorphic region of the *EF1α* gene, followed by Illumina Miseq sequencing of the amplicons and analysis of the reads to identify *Fusarium* species. A new specific PCR primer pair was designed that targets the *EF1α* gene, allowing species-level discrimination, which confirms the potential of this gene previously reported [27]. In metabarcoding techniques, the initial PCR is an important step to correctly describe a community due to bias resulting from primer mismatches or PCR inhibitors [42]. As we selected conserved primers preferentially targeting the *EF1α* gene from species that belong to a single genus, *Fusarium*, primer mismatch should be avoided. An advantage of using the *EF1α* gene to study *Fusarium* communities is that it appears to be present as a single copy in this genus [27], thus circumventing problems caused by the occurrence of non-orthologous copies or a variable copy number among species, like in the case of the *ITS* gene, which is mainly used for metabarcoding [18]. Recently specific primers for the *EF1α* gene were used to assess *Fusarium* diversity in field samples from soil and wheat kernels in association with 454 pyrosequencing [24]. In a different study [22], the authors used another single-copy protein-coding locus (RPB2) to target the genus *Fusarium* associated with the rhizosphere of native grassland plants using 454 technology [22]. In our study, Illumina Miseq technology was used in order to deepen the sequencing compared to 454 technology. In addition, Illumina technology enabled the simultaneous processing of a larger number of samples. Indeed, in our study, up to 154 field samples could be sequenced at the same time, with a mean number of 88,663 reads per sample before bioinformatic analysis.

DNA mock communities and artificially infected grain samples were used to validate our metabarcoding protocol. A sufficient number of reads could be retrieved and assigned to species in our DNA mock communities using the forward Illumina MiSeq sequences. Reverse Illumina MiSeq sequences could have given complementary information, but could not be used due to poor quality. One difficulty using high-throughput sequencing technologies is to validate OTUs with low numbers of reads, as they can be the result of PCR and/or sequencing errors [43]. DNA mock communities were used to determine an error rate, which was calculated by comparing the Sanger sequence of each strain included in each mock community with the sequence of the OTUs obtained. The maximum error rate for the 5 mock communities was estimated to be 0.3%. Thus, to ensure that correct OTUs were retained in all samples, all OTUs with a number of reads below 0.3% of the total reads per sample were removed. Rare OTUs were thus removed from all samples with a similar rule in order to avoid overestimation of the diversity present in the samples. *EF1α* sequences from all 20 *Fusarium* species used were successfully retrieved after analysis of the DNA mock communities. In addition, 3 species out of 10 in the FGSC (*F*. *cortaderiae*, *F*. *meridionale* and *F*. *acaciae-mearnsii*) could also be identified using our protocol. Interestingly, even though absolute quantification is not possible due to PCR bias, all *Fusarium* species in mock communities 2 and 4 yielded approximately identical percentages of total reads. This might be due to the fact that *EF1α* used for barcoding is a single copy gene. However, the amplification efficiency of the primers developed in this study might be different and may depend on the target species. In mock community 3, *F*. *culmorum* accounted for only 5% of the reads.

All *Fusarium* species, except *F*. *culmorum*, could be detected in the artificially infected grain samples. Several markers of the strain used for inoculation were sequenced and the sequences obtained were identical to the sequences of other *F*. *culmorum* strains. However, *F*. *culmorum* was detected in 24 field samples showing that our metabarcoding protocol is also able to specifically identify *F*. *culmorum* in complex samples. A close look at the results for artificially infected grain samples showed that the OTU assigned to *F*. *graminearum* had a score of 56. This score is a bootstrap value; the higher it is, the greater the probability that the correct name is given to an OTU. The other OTUs had a score of 75–100. After careful checking, some reads grouped in this OTU could be assigned to *F*. *culmorum*. The errors seemed to be linked to a PCR chimera between *F*. *graminearum* and *F*. *culmorum* not recognized by the bioinformatic pipeline. However, careful examination of the final identification scoring should prevent these errors.

The analysis of a sample using our metabarcoding approach will yield a large batch of barcode sequences. Assignation to a species name using a barcode sequence will first depend on the quality of the reference database used and second on the phylogenic resolution of the chosen barcode. The phylogenic resolution of the *EF1α* barcode used in this publication allowed us to identify species commonly present in FHB disease. However some closely related (or cryptic-) species might be difficult to differentiate due to barcode resolution. For example, to the *EF1α* region that was used here will not enable to discriminate some strains of the FGSC complex (*F*. *vorosii*, *F*. *brasilicum*, *F*. *gerlachii*) from *F*. *graminearum*. Another example are the closely related species *F*. *sibiricum* and *F*. *sporotrichioide*s [44]. It is obvious that the quality of the overall assignation pipeline will depend on the quality of the reliability of the DNA sequence database used, and on the fact that the species names are regularly updated according to the state of the art regarding taxonomy in this intensively studied genus.

As the protocol proposed was aimed at assessing *Fusarium* diversity on field samples, it was important to test the reproducibility and the sensitivity of the technique. In this study, metabarcoding proved to be reproducible using DNA mock community samples, independent of the amplification or the sequencing run. Numbers of PCR cycles have been reported as an important factor increasing contamination [45], but no such effects were observed in our experiments by testing different cycle numbers. Analysis of artificially infected wheat samples showed the high sensitivity of metabarcoding for *Fusarium*. One infected grain could be detected in a total of 10,000 grains.

As a proof of principle, further analyses were performed on 65 cereal samples harvested across the major cereal-growing regions of France. Overall, 17 different *Fusarium* species were detected in these samples of barley, durum and soft wheat. In wheat samples, *F*. *graminearum* and *F*. *poae* were the dominant species, followed by *Fusarium sp*. *(FTSC)*, *F*. *culmorum* and *F*. *avenaceum*, which are common *Fusarium* species previously recovered from wheat in France [2, 3].In barley samples, *F*. *poae* and *Fusarium sp*. *(FTSC)* were the dominant species, followed by *F*. *langsethiae*, *F*. *graminearum* and *F*. *avenaceum*. These species were previously reported from barley samples collected in France, except *F*. *langsethiae* [2, 3]. As *F*. *langsethiae* was described in 2004 [46] and it resembles *F*. *poae* morphologically, *F*. *langsethiae* was probably present on barley sampled in France in 2004 but was likely assigned to *F*. *poae* in a previous survey [2]. To our knowledge, this is the first time species such as *F*. *thapsinum*, *F*. *torulosum*, *F*. *redolens* and *F*. *mahasenii* have been reported on barley, and *F*. *proliferatum* on wheat collected in France. In a survey conducted on FGSC diversity in France in 2011, *F*. *graminearum sensu stricto* was predominant on wheat, barley and maize, but three isolates of *F*. *cortaderiae* and two isolates representing *F*. *graminearum* × *F*. *boothii* hybrids were also identified from maize [47]. The tool developed in this study should recover the *F*. *cortaderiae* isolates in France but not the hybrid. As the symptoms on cereal grains are not specific to the *Fusarium* species causing the disease, real-time PCR tests have been developed for *Fusarium* species detection and quantification in field samples [15]. Some of these tests are multiplexed in order to reduce the costs of analysis [48, 49]. Using our metabarcoding protocol, up to 12 *Fusarium* species could be detected in field samples and up to 150 samples could be processed at the same time. Species like *F*. *torulosum*, *F*. *sambucinum* and 3 species of the FGSC were also detected. As far as we know, no specific real-time PCR tests are available for these species. It is difficult to compare real-time PCR tests with the metabarcoding technique to detect *Fusarium* species because the efficiency of the species-specific primers used in real-time PCR tests and the length of the amplified fragment vary depending on the species. We used the real-time PCR only to verify that *Fusarium* species were present in our field samples. Discrepancies were observed between the results of the two techniques, with up to 37% non-corresponding detection observed (Table 4). In the majority of cases, discrepancies were due to detection of species by metabarcoding but not by real-time PCR. It is difficult to conclude whether these correspond to true or false positives. Due to PCR amplification biases, each technique may be favourable to the detection of one species rather than another. A possible explanation for these discrepant results may be that the limit of detection of each test (metagenomics and the different species-specific qPCR test) may be variably affected by the presence of inhibiting compounds in the DNA extracts. Since the level of contamination by some of the *Fusarium* species was sometimes very low, it is likely that some of the analyses were at the limit of detection for some of the targets.

Some of the reads generated were not assigned to species of *Fusarium*. As the identification of other genera was not included in the objectives of this study, the corresponding OTUs were only assigned to the genus level even though a species name could be determined. Importantly, when low *Fusarium* contamination was found in field samples, the amounts of non-*Fusarium* species increased. It is also interesting to note that in artificially infected samples, when *Fusarium* contamination decreased, an increase in non-*Fusarium* species was observed. This could be due to the lack of target species that decrease competition for the primer, leading to less specific amplification. However, it was shown that reagent and laboratory contamination increased in the results, especially when there was a low amount of starting material [50]. As a result, special care should be taken when performing metabarcoding experiments.

## Conclusion

Using mock communities and artificially infected samples, a protocol was developed to assess the composition of *Fusarium* species in field samples without *a priori* criteria. For the first time, the sensitivity and reproducibility of the technique proved to be suitable to detect most of the *Fusarium* species with low contamination levels (up to 1 seed in 10,000). Moreover, 3 species out of the 10 tested in the FGSC could be distinguished. The possibility of multiplexing samples makes this protocol suitable for large-scale *Fusarium* epidemiological surveys, such as those studying the impact of climate change, or of evolving cultural farming practices.

## Supporting information

S1 Fig Strain used in this study. (PDF)

S2 Fig Phylogenetic tree using EF1α sequences. (PDF)

S3 Fig Phylogenetic tree using RPB2 sequences. (PDF)

S4 Fig Description of field samples. Sampling year, variety treatment, cultural practices and location are presented for each sample.
(PDF)

S5 Fig Test of primers’ specificity. This file summarizes the fungal species that were tested and detected by the PCR primer proposed in this publication. After PCR, the results were visualised on an agarose gel. D represent a faint band on the gel, + means detected and–means not detected.
(PDF)

S6 Fig Position of the primers on EF1α sequence. (PDF)

S7 Fig Description of the pipeline for Miseq Illumina reads analysis. (PDF)

S1 Table Real-time PCR results of fields samples analysis. (XLSX)
