# Supplementary material for: Metabarcoding targeting the EF1 alpha region to assess Fusarium diversity on cereals
Source: PLoS One. 2019 Jan 11;14(1):e0207988. doi: 10.1371/journal.pone.0207988 (PMC6329491; doi:10.1371/journal.pone.0207988)
Supplement: S1 Fig — (PDF) [file pone.0207988.s001.pdf]

Species	internal lab id	usage in the publication	initial collection	R-Syst::fungi id number of the sequences	origine	isolate source
Alternaria alternata	C61	Mock1	UMR BIOGER, INRA, Grignon, France	AG-C61-ITS1F.G03		Brassica napus
Botrytis cinerea	C87	Mock1	UMR BIOGER, INRA, Grignon, France	AG-C87-ITS1F.A06		Brassica napus
Calonectria pseudonaviculata	93	Mock3	UMR BIOGER, INRA, Grignon, France	AG-93-ITS1F.G12		Buxus
Cladosporium delicatulum	C58	Mock2	UMR BIOGER, INRA, Grignon, France	AG-C58-ITS1F.F03, AG-02-Q-C58-PYROFUS-F2.B0		Brassica napus
Cladosporium sphaerospermum	96	Mock2	UMR BIOGER, INRA, Grignon, France	AG-96-PYROFUS-F2.H02		Lawn
Colletotrichum orbiculare	C-orbiculare	Mock2	UMR BIOGER, INRA, Grignon, France	AG-C. graminicola-PYROFUS-F2.H02		Zea mays
Epicoccum nigrum	100	Mock3	UMR BIOGER, INRA, Grignon, France	AG-colleto-ITS1F.C04, AG-C. graminicola-PYROFUS-F2.H02		Triticum aestivum
Eutypa lata	0002-11	Mock2	UMR BIOGER, INRA, Grignon, France	AG-11-Bor-ITS1F.C10, AG-Eutypa-lata-EF1Fungus-sppR.C0		Vitis vinifera
Fusarium acaciae meamsii	NRRL26755	Mock5	USDA ARS, Peoria, Illinois, USA	AG-LSV834-ITS1F.D09, AG-LSV834-PYROFUS-F2.D0		
Fusarium aethipicum	NRRL46722	Mock5	USDA ARS, Peoria, Illinois, USA	AG-LSV835-ITS1F.E09, AG-LSV835-PYROFUS-F2.C09		
Fusarium asiaticum	NRRL138181	Mock5	USDA ARS, Peoria, Illinois, USA	AG-LSV832-ITS1F.C10, AG-LSV832-PYROFUS-F2.B0		
Fusarium austroamericanum	NRRL28728	Mock5	USDA ARS, Peoria, Illinois, USA	AG-LSV838-ITS1F.E10, AG-LSV838-PYROFUS-F2.H05		
Fusarium avenaceum	MTK0070	Mock4	Mycotek, FSOV project, Arvalis institut du végétal, Grignon, France	AG-MTK0070-ITS1F.F06; AG-MTK0070-PYROFUS-F2.E01, MTK0070_RPB	South west, France	
Fusarium avenaceum	Fa	Infected grains F8 series		NS		
Fusarium brasilicum	NRRL31238	Mock5	USDA ARS, Peoria, Illinois, USA	AG-LSV840-ITS1F.G10, AG-LSV840-PYROFUS-F2.B06		
Fusarium cortaderiae	NRRL31171	Mock5	USDA ARS, Peoria, Illinois, USA	AG-LSV839-ITS1F.F10, AG-LSV839-PYROFUS-F2.A0		
Fusarium culmorum	Fc17	Infected grains F8 series	UMR BIOGER, INRA, Grignon, France	B7_SI00359, B7_SI00358_17		Triticum aestivum
Fusarium culmorum	MTK0055	Mock3	Mycotek, FSOV project, Arvalis institut du végétal, Grignon, France	AG-MTK0055-ITS1F.E06, AG-MTK0055-PYROFUS-F2.A01	Oise (60), France	Triticum aestivum (var. Isengrain)
Fusarium culmorum	MTK0009	Mock4	Mycotek, FSOV project, Arvalis institut du végétal, Grignon, France	AG-MTK0009-ITS1F.B06, AG-MTK0009-Fc-PYROFUS-F2.A11, MTK0009_RPB	Charente (16), France	Triticum aestivum
Fusarium equiseti (FIESC 14)	C33	Mock2	UMR BIOGER, INRA, Grignon, France	AG-C33-ITS1F.G01, AG-C33-EF1FusaBiogerF.B04, C33_RPB2		Brassica napus
Fusarium gerlachii	NRRL38380	Mock5	USDA ARS, Peoria, Illinois, USA	AG-LSV831-ITS1F.B10, AG-LSV831-PYROFUS-F2.A05		
Fusarium graminearum	FG20	Infected grains F8 series	UMR BIOGER, INRA, Grignon, France	A10_SI00359, A10_SI00358_17		Triticum aestivum
Fusarium graminearum	MTK0019	Mock2	Mycotek, FSOV project, Arvalis institut du végétal, Grignon, France	AG-MTK0019-ITS1F.B03, AG-16-Q-MTK0019-PYROFUS-F2.D0	Tarn et Garonne, France	Zea mays
Fusarium graminearum	LSV M808	Infected grains G10PxLxTx series	ANSES laboratoire de pathologie végétale, Nancy, France	NS	Drôme (26), France	Triticum aestivum (seeds)
Fusarium langsethiae	Fl39	Mock4	UMR BIOGER, INRA, Grignon, France	AG-Fl39-ITS1F.A08, AG-Fl39-PYROFUS-F2.D02, Fl39_RPB2	East, France	Triticum aestivum (seeds)
Fusarium langsethiae	FL33	Infected grains F8 series	UMR BIOGER, INRA, Grignon, France	E3_SI00359, E3_SI00358_17		Triticum aestivum (seeds)
Fusarium langsethiae	LSV M846	Infected grains G10PxLxTx series	ANSES laboratoire de pathologie végétale, Nancy, France	NS	NA	Barley
Fusarium meridionale	NRRL29010	Mock5	USDA ARS, Peoria, Illinois, USA	AG-LSV841-ITS1F.H10, AG-LSV841-PYROFUS-F2.C06		
Fusarium mesoamericanum	NRRL25797	Mock5	USDA ARS, Peoria, Illinois, USA	AG-LSV837-ITS1F.D10, AG-LSV837-PYROFUS-F2.G05		
Fusarium oxysporum	FOR 4	Mock1	C. Alabouvette (MIAE?)	AG-FOR4-ITS1F.D06, AG-FOR4-EF1FusaBiogerF.D02	France	Raphanus sativus
Fusarium poae	FP1	Infected grains F8 series	UMR BIOGER, INRA, Grignon, France	C5_SI00359, C5_SI00358_17		Triticum aestivum (seeds)
Fusarium poae	LSV M861	Infected grains G10PxLxTx series	ANSES laboratoire de pathologie végétale, Nancy, France	NS	Puy-de-Dôme (63), France	Triticum aestivum (seeds)
Fusarium poae	MTK0005	Mock3	Mycotek, FSOV project, Arvalis institut du végétal, Grignon, France	HB_seq_03, HB_EF1_03, MTK0005_RPB2	Boigneville, France	
Fusarium pseudonygamai	FoxFMO14	Mock3	UMR BIOGER, INRA, Grignon, France	NS		
Fusarium sambucinum	C39	Mock1, 2, 3, 4	UMR BIOGER, INRA, Grignon, France	AG-C39-ITS1F.A03, AG-C39-EF1FusaBiogerF.D04		Brassica napus
Fusarium sp. (FSSC 11)	FJMO	Mock1	UMR BIOGER, INRA, Grignon, France	AG-FJMOxxx-ITS1F.H06, AG-FJMO_-EF1FusaBiogerF.H02 , FJMO_RPB2		
Fusarium sp.	22374	Mock1	USDA ARS, Peoria, Illinois, USA	AG-22374-ITS1F.A12, AG-22374-EF1FusaBiogerF.B01	Germany	Soil
Fusarium sporotrichioïdes	Fs23	Mock3	UMR BIOGER, INRA, Grignon, France	AG-Fs23-ITS1F.G06, AG-Fs23-PYROFUS-F2.C02 , Fs23_RPB	South West, France	Triticum aestivum (seeds)
Fusarium staphyleae	22316	Mock1	USDA ARS, Peoria, Illinois, USA	AG-22316-ITS1F, AG-22316-EF1FusaBiogerF.C01	New Jersey, USA	Staphylea trifolia
Fusarium subglutinans	Fsub69	Mock4	MIAE, INRA, Dijon, France	AG-16-Q-Fsub69-LEPTOLIFE-ITS1F.F08, AG-17-Q-Fsub69-PYROFUS-F2.E02, Fsub69_RPB		
Fusarium subglutinans	FU12238	Infected grains F8 series	Arvalis institut du vegétal, Grignon, France	NS		
Fusarium temperatum	Fpro32-2	Mock4	MIAE, INRA, Dijon, France	AG-Fpro32-2-ITS1F.B08, AG-Fpro32-2-PYROFUS-F2.A02_141029105F, Fpro32-2_RPB		
Fusarium sp. (FTSC)	Ft12	Mock4, infected grains F8 series	UMR BIOGER, INRA, Grignon, France	AG-Ft12-ITS1F.D08, AG-Ft12-PYROFUS-F2.B02		Triticum aestivum (seeds)
Fusarium tricinctum	LSV M860	Infected grains G10PxLxTx series	ANSES laboratoire de pathologie végétale, Nancy, France	NS	Eure-et-Loir (28), France	Triticum aestivum (seeds)
Fusarium verticillioides	Fmoni62	Mock4	MIAE, INRA, Dijon, France	AG-FMoni62-ITS1F.C08, AG-20-Q-Fmoni62-PYROFUS-F2.G02, Fmoni62_RPB		
Fusarium verticillioides	FU09041	Infected grains F8 series	Arvalis institut du vegétal, Grignon, France	NS		
Fusarium vorosii	NRRL38208	Mock5	USDA ARS, Peoria, Illinois, USA	AG-LSV833-ITS1F.C09, AG-LSV833-PYROFUS-F2.C0		
Gaeumannomyces graminis var. tritici	MTK0077	Mock2	Mycotek, FSOV project, Arvalis institut du végétal, Grignon, France	AG-MTK0077_F05		
Helminthosporium tritici-repentis	MTK0038	Mock1	Mycotek, FSOV project, Arvalis institut du végétal, Grignon, France	AG-MTK0038_E05, HB_EF1_23	Prosnes (51), France	Triticum aestivum seeds (var. Caphorn)
Leptosphaeria biglobosa	C68	Mock4	UMR BIOGER, INRA, Grignon, France	AG-C68-ITS1F.B05, AG-04-ssQ-C68-PYROFUS-F2.A0		Brassica napus
Leptosphaeria maculans	102	Mock3	UMR BIOGER, INRA, Grignon, France	AG-102-ITS1F.B07, AG-102-PYROFUS-F2.F02		Brassica napus
Microdochium bolleyi	98	Mock4	UMR BIOGER, INRA, Grignon, France	AG-98-ITS1F.F06, AG-98-PYROFUS-F2.E01		Triticum aestivum
Microdochium majus	MTK0025	Mock3	Mycotek, FSOV project, Arvalis institut du végétal, Grignon, France	AG-MTK0025-ITS1F.C06, AG-MTK0025-PYROFUS-F2.B0	Loire-Atlantique (44), France	Triticum aestivum (seeds)
Microdochium nivale	Mn08N58	Mock2	UMR BIOGER, INRA, Grignon, France	NS		
Mortierella elongata	95	Mock3	UMR BIOGER, INRA, Grignon, France	AG-95-ITS1F.C06		Lawn
Phaeoacremonium mortoniae	0002-12	Mock1	UMR BIOGER, INRA, Grignon, France	AG-12-Bor-ITS1F.D10,AG-Phaeoacremonium-mortoniae-EF1Fungus-sppR.E03		Vitis vinifera
Parastagonospora nodorum	MTK0081	Mock1	Mycotek, FSOV project, Arvalis institut du végétal, Grignon, France	AG-MTK0081_G05_ITS1F, AG-MTK0081-PYROFUS-EF1-F1.F02		
Pseudocercosporella herpotrichoïdes	MTK0049	Mock2	Mycotek, FSOV project, Arvalis institut du végétal, Grignon, France	AG-MTK0049-ITS1F.C03, AG-MTK0049-PYROFUS-F2.D01		
Puccinia triticina	B77Saba	Mock1	UMR BIOGER, INRA, Grignon, France	B77Saba_ITS1F		
Sclerotinia sclerotiorum	C62	Mock1	UMR BIOGER, INRA, Grignon, France	AG-C62-ITS1F.H03, AG-C62-PYROFUS-F2.G01		Brassica napus
Pseudonectria buxi	94	Mock3	UMR BIOGER, INRA, Grignon, France	AG-94-ITS1F.B06, AG-94-PYROFUS-F2.G02		Buxus
Zymoseptoria tritici	MTK0030	Mock4	Mycotek, FSOV project, Arvalis institut du végétal, Grignon, France	AG-MTK0030-ITS1F.D06; AG-MTK0030-PYROFUS-F2.C	Bignan (56), France	Triticum aestivum seeds (var. premio)

NS: sequences not availiable
