# Supplementary figures and images for: Metabarcoding targeting the EF1 alpha region to assess Fusarium diversity on cereals
Source: PLoS One. 2019 Jan 11;14(1):e0207988. doi: 10.1371/journal.pone.0207988 (PMC6329491; doi:10.1371/journal.pone.0207988)

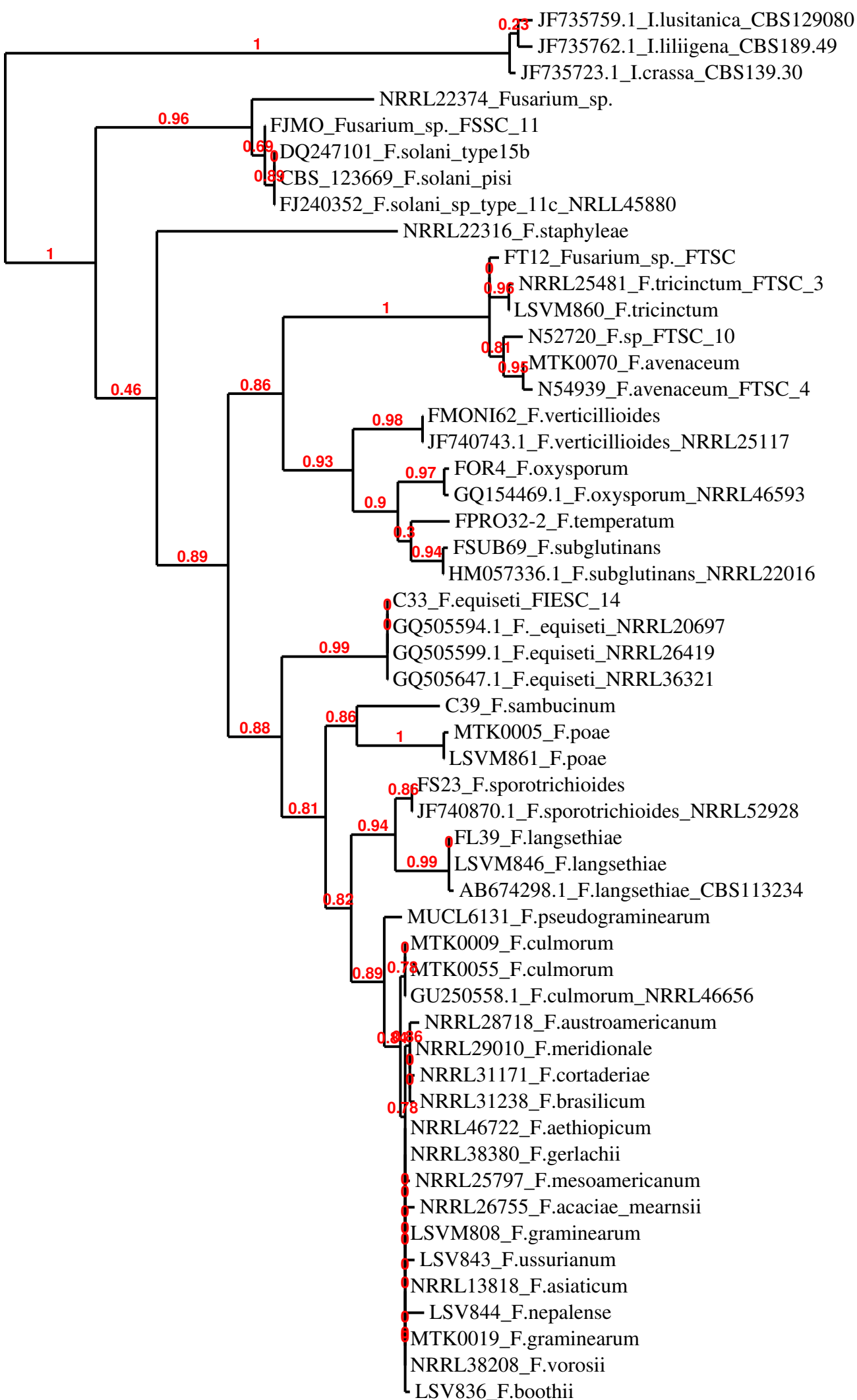

0.1

Supplement: S2 Fig — (PDF) [file pone.0207988.s002.pdf]

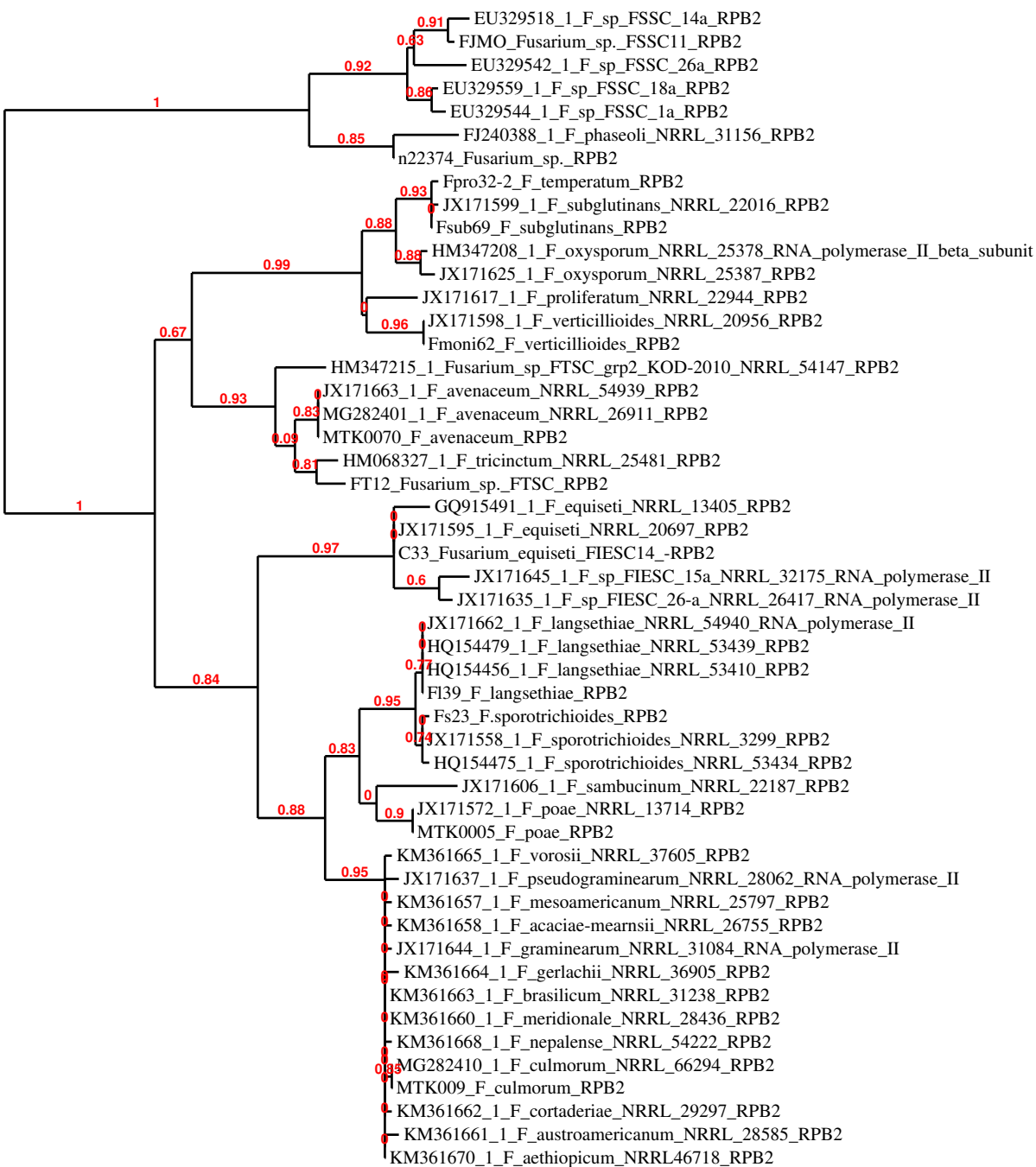

Supplement: S3 Fig — (PDF) [file pone.0207988.s003.pdf]
