# Supplementary material for: Metabarcoding targeting the EF1 alpha region to assess Fusarium diversity on cereals
Source: PLoS One. 2019 Jan 11;14(1):e0207988. doi: 10.1371/journal.pone.0207988 (PMC6329491; doi:10.1371/journal.pone.0207988)
Supplement: S4 Fig — Sampling year, variety treatment, cultural practices and location are presented for each sample. (PDF) [file pone.0207988.s004.pdf]

Sample name	Cereal	Sampling year	Variety	Crop rotation	Cultural practices	Treatment	Location
S_2721	Durum wheat	2014	KARUR	Sunflower	NO PLOWING	ND	PERIGNY (17)
S_2722	Durum wheat	2014	MIRADOUX	Sunflower	NO PLOWING	ND	MONTASTRUC-LA-CONSEILLERE (31)
S_2723	Durum wheat	2014	KARUR	Potato	ND	ND	VILLAMBLAIN (45)
S_2724	Durum wheat	2014	PLUSSUR	Rapeseed	PLOWING	ND	PUERTHE (28)
S_2725	Durum wheat	2014	KARUR	Maize	PLOWING	ND	IGNEVILLE (28)
S_2726	Durum wheat	2014	ATOUDUR	Sunflower	NO PLOWING	ND	MONTESQUIEU-LAURAGAIS (31)
S_2727	Durum wheat	2014	MIRADOUX	Sunflower	NO PLOWING	ND	PAIZAY-LE-SEC (86)
S_2728	Durum wheat	2014	TABLUR	Sunflower	NO PLOWING	ND	THURE (86)
S_2729	Durum wheat	2014	MIRADOUX	Maize	PLOWING	ND	PORTES-LES-VALENCE (26)
S_2730	Durum wheat	2014	MIRADOUX	Rapeseed	NO PLOWING	ND	SAINT CLOUD (45)
S_2731	Durum wheat	2014	PLUSSUR	Rapeseed	PLOWING	ND	EPIEDS-EN-BEAUCE (45)
S_2732	Durum wheat	2014	SCULPTUR	Durum wheat	PLOWING	ND	TRIAIZE (85)
S_2733	Bread wheat	2014	COURTOT	Sugarbeet	PLOWING	ND	ROISNEY SAINT DENIS (28)
S_2734	Bread wheat	2014	ACCROC	Sunflower	DIRECT SAWING	ND	SAINT LACTENCIN (36)
S_2735	Bread wheat	2014	EUCLIDE	Maize	DIRECT SAWING	ND	MAZE (49)
S_2736	Bread wheat	2014	CELLULE	Rapeseed	PLOWING	ND	NA (72)
S_2737	Bread wheat	2014	EXPERT	Sugarbeet	PLOWING	ND	NOREUIL (62)
S_2738	Bread wheat	2014	RUBISKO	Maize	PLOWING	ND	LE MERRE (60)
S_2739	Bread wheat	2014	PALEDOR	Maize	PLOWING	ND	PLOUMOGUER (29)
S_2740	Barley	2014	SEBASTIAN	Bread wheat	PLOWING	ND	TOURNOISIS (45)
S_2741	Barley	2014	SEBASTIAN	Rapeseed	SUPERFICIAL PLOWING	ND	ALVIMORE (76)
S_2742	Barley	2014	ETINCEL	Bread wheat	SUPERFICIAL PLOWING	ND	FOUCHERES (10)
S_2425	Bread wheat	2015	Bermude	Fava bean (maize stubble brought)	SUPERFICIAL PLOWING	Untreated	BOIGNEVILLE (91)
S_2426	Bread wheat	2015	Bermude	Fava bean (maize stubble brought)	SUPERFICIAL PLOWING	Treated	BOIGNEVILLE (91)
S_2427	Bread wheat	2015	Bermude	Fava bean (maize stubble brought)	SUPERFICIAL PLOWING	Treated	BOIGNEVILLE (91)
S_2428	Bread wheat	2015	Bermude	Fava bean (maize stubble brought)	SUPERFICIAL PLOWING	Treated	BOIGNEVILLE (91)
S_2429	Bread wheat	2015	Bermude	Fava bean (maize stubble brought)	SUPERFICIAL PLOWING	Treated	BOIGNEVILLE (91)
S_2430	Bread wheat	2015	Bermude	Fava bean (maize stubble brought)	SUPERFICIAL PLOWING	Treated	BOIGNEVILLE (91)
S_2431	Bread wheat	2015	Bermude	Fava bean (maize stubble brought)	SUPERFICIAL PLOWING	Treated	BOIGNEVILLE (91)
S_2432	Bread wheat	2015	Bermude	Fava bean (maize stubble brought)	SUPERFICIAL PLOWING	Treated	BOIGNEVILLE (91)
S_2433	Bread wheat	2015	Bermude	Fava bean (maize stubble brought)	SUPERFICIAL PLOWING	Treated	BOIGNEVILLE (91)
S_2434	Bread wheat	2015	Bermude	Fava bean (maize stubble brought)	SUPERFICIAL PLOWING	Treated	BOIGNEVILLE (91)
S_2435	Bread wheat	2015	Bermude	Fava bean (maize stubble brought)	SUPERFICIAL PLOWING	Treated	BOIGNEVILLE (91)
S_2436	Bread wheat	2015	Bermude	Fava bean (maize stubble brought)	SUPERFICIAL PLOWING	Treated	BOIGNEVILLE (91)
S_2437	Bread wheat	2015	Bermude	Fava bean (maize stubble brought)	SUPERFICIAL PLOWING	Treated	BOIGNEVILLE (91)
S_2438	Bread wheat	2015	Bermude	Fava bean (maize stubble brought)	SUPERFICIAL PLOWING	Treated	BOIGNEVILLE (91)
S_948	Bread wheat	2015	PR22R58	Maize	SUPERFICIAL PLOWING	Treated	BERGERAC (24)
S_949	Bread wheat	2015	PR22R58	Maize	SUPERFICIAL PLOWING	Treated	BERGERAC (24)
S_950	Bread wheat	2015	PR22R58	Maize	SUPERFICIAL PLOWING	Treated	BERGERAC (24)
S_951	Bread wheat	2015	PR22R58	Maize	SUPERFICIAL PLOWING	Treated	BERGERAC (24)
S_952	Bread wheat	2015	PR22R58	Maize	SUPERFICIAL PLOWING	Untreated	BERGERAC (24)
S_953	Bread wheat	2015	PR22R58	Maize	SUPERFICIAL PLOWING	Treated	BERGERAC (24)
S_954	Bread wheat	2015	PR22R58	Maize	SUPERFICIAL PLOWING	Treated	BERGERAC (24)
S_955	Bread wheat	2015	PR22R58	Maize	SUPERFICIAL PLOWING	Treated	BERGERAC (24)
S_956	Bread wheat	2015	PR22R58	Maize	SUPERFICIAL PLOWING	Treated	BERGERAC (24)
S_5255	Barley	2015	Sebastian	Fava bean (maize stubble brought)	SUPERFICIAL PLOWING	Treated	BOIGNEVILLE (91)

S_5256	Barley	2015	Sebastian	Fava bean (maize stubble brought)	SUPERFICIAL PLOWING	Treated	BOIGNEVILLE (91)
S_5257	Barley	2015	Sebastian	Fava bean (maize stubble brought)	SUPERFICIAL PLOWING	Treated	BOIGNEVILLE (91)
S_5258	Barley	2015	Sebastian	Fava bean (maize stubble brought)	SUPERFICIAL PLOWING	Treated	BOIGNEVILLE (91)
S_5259	Barley	2015	Sebastian	Fava bean (maize stubble brought)	SUPERFICIAL PLOWING	Treated	BOIGNEVILLE (91)
S_5260	Barley	2015	Sebastian	Fava bean (maize stubble brought)	SUPERFICIAL PLOWING	Treated	BOIGNEVILLE (91)
S_5261	Barley	2015	Sebastian	Fava bean (maize stubble brought)	SUPERFICIAL PLOWING	Treated	BOIGNEVILLE (91)
S_5351	Barley	2015	KWS Irina	Maize	SUPERFICIAL PLOWING	Untreated	LIMETZ VILLEZ (78)
S_5352	Barley	2015	KWS Irina	Maize	SUPERFICIAL PLOWING	Treated	LIMETZ VILLEZ (78)
S_5353	Barley	2015	KWS Irina	Maize	SUPERFICIAL PLOWING	Treated	LIMETZ VILLEZ (78)
S_5354	Barley	2015	KWS Irina	Maize	SUPERFICIAL PLOWING	Treated	LIMETZ VILLEZ (78)
S_5355	Barley	2015	KWS Irina	Maize	SUPERFICIAL PLOWING	Treated	LIMETZ VILLEZ (78)
S_5356	Barley	2015	KWS Irina	Maize	SUPERFICIAL PLOWING	Treated	LIMETZ VILLEZ (78)
S_5359	Barley	2015	KWS Irina	Maize	SUPERFICIAL PLOWING	Treated	LIMETZ VILLEZ (78)
S_5426	Barley	2015	Sebastian	Durum wheat (maize stubble brought)	SUPERFICIAL PLOWING	Treated	PRENOUVELLON (41)
S_5427	Barley	2015	KWS Irina	Durum wheat (maize stubble brought)	SUPERFICIAL PLOWING	Treated	PRENOUVELLON (41)
S_5428	Barley	2015	RGT Planet	Durum wheat(maize stubble brought)	SUPERFICIAL PLOWING	Treated	PRENOUVELLON (41)
S_5429	Barley	2015	Sebastian	Durum wheat(maize stubble brought)	SUPERFICIAL PLOWING	Treated	PRENOUVELLON (41)
S_5430	Barley	2015	KWS Irina	Durum wheat(maize stubble brought)	SUPERFICIAL PLOWING	Treated	PRENOUVELLON (41)
S_5431	Barley	2015	RGT Planet	Durum wheat(maize stubble brought)	SUPERFICIAL PLOWING	Treated	PRENOUVELLON (41)
