# Supplementary material for: Metabarcoding targeting the EF1 alpha region to assess Fusarium diversity on cereals
Source: PLoS One. 2019 Jan 11;14(1):e0207988. doi: 10.1371/journal.pone.0207988 (PMC6329491; doi:10.1371/journal.pone.0207988)
Supplement: S5 Fig — This file summarizes the fungal species that were tested and detected by the PCR primer proposed in this publication. After PCR, the results were visualised on an agarose gel. D represent a faint band on the gel, + means detected and–means not detected. (PDF) [file pone.0207988.s005.pdf]

Species	ID	Detection
Alternaria alternata	C61	D
Botrytis sp.	C87	D
Calonectria pseudonaviculata	93	-
Cladosporium sphaerospermum	96	-
Cladosporium sp.	C58	**+**
Colletrotrichum sp.	CG	**+**
Eutypa lata	E. lata	**+**
Epicoccum nigrum	100	**+**
Fusarium acaciae mearnsii	**NRRL 26755**	**+**
Fusarium aethiopicum	**NRRL 46722**	**+**
Fusarium asiaticum	**NRRL 13818**	**+**
Fusarium austroamericanum	**NRRL 28718**	**+**
Fusarium avenaceum	**MTK0070**	**+**
Fusarium boothii	**NRRL 29011**	**+**
Fusarium brasilicum	**NRRL 31238**	**+**
Fusarium cortaderiae	**NRRL 31171**	**+**
Fusarium culmorum	**MTK0009**	**+**
Fusarium culmorum	**MTK0055**	**+**
Fusarium equiseti (FIESC14)	**C33**	**+**
Fusarium gerlachii	**NRRL 38380**	**+**
Fusarium graminearum	**MTK0019**	**+**
Fusarium langsethiae	**Fl39**	**+**
Fusarium meridionale	**NRRL 29010**	**+**
Fusarium mesoamericanum	**NRRL 25797**	**+**
Fusarium nepalense	**NRRL 54222**	D
Fusarium oxysporum	**FOR 4**	**+**
Fusarium poae	**MTK0005**	**+**
Fusarium pseudonygamai	**FoxFMO14**	**+**
Fusarium sambucinum	**C39**	**+**
Fusarium sp. (FSSC11)	**FJMO**	**+**
Fusarium sp.	**22374**	**+**
Fusarium sporotrichioïdes	**Fs23**	**+**
Fusarium staphyleae	**22316**	**+**
Fusarium subglutinans	**Fsub69**	**+**
Fusarium temperatum	**Fpro32-2**	**+**
Fusarium sp. (FTSC)	**FT12**	**+**
Fusarium ussurianum	**LSV843**	**+**
Fusarium verticillioides	**Fmoni62**	**+**
Fusarium vorosii	**NRRL 38208**	**+**
Gaeumannomyces graminis var. tritici	MTK0077	-
Helminthosporium tritici-repentis	MTK0038	-
Leptosphaeria biglobosa	C68	-
Leptosphaeria maculans	102	**+**
Microdochium bolleyi	98	-
Mortierella elongata	95	-
Microdochium majus	MTK0025	-
Microdochium nivale	Mn08N58	-
Volutella ciliata	94	-
Pseudocercosporella herpotrichoïdes	MTK0049	**+**
Phaeoacremonium mortoniae	P. mortoniae	**+**
Puccinia triticina	B77Saba	-
Parastagonospora nodorum	MTK0081	-
Sclerotinia sclerotiorum	C62	D
Zymoseptoria tritici	MTK0030	**+**

D detected faint band on gel
\+ band on gel
\- no band on gel
