# Supplementary material for: Metabarcoding targeting the EF1 alpha region to assess Fusarium diversity on cereals
Source: PLoS One. 2019 Jan 11;14(1):e0207988. doi: 10.1371/journal.pone.0207988 (PMC6329491; doi:10.1371/journal.pone.0207988)
Supplement: S6 Fig — (PDF) [file pone.0207988.s006.pdf]

>JF740867.1 Fusarium graminearum strain NRRL 52799 translation elongation factor alpha gene, partial cds

EF1-F2

GAGAAGACTCACCTTAACGTCGTCGTCATCGGCCACGTCGACTCTGGCAAGTCGACCACTGTGAGTACCACCG

CATCCCAACCCCGCCGACACTTGGCG**GGGTAGTTTCAAATTTCCAATGTGCTGACATACTTTGATAGACCGGT**

CACTTGATCTACCAGTGCGGTGGTATCGACAAGCGAACCATCGAGAAGTTCGAGAAGGTTGGTCTCATTTTCC

TCGATCGCGCGCCCTTTTCCCTTTCGAAATATCATTCGAATCGCCCTCACACGACGACTCGATACGCGCCTGT

TACCCCGCTCGAGGTCAAAAATTTTGCGGCTTTGTCGTAATTTTTTTCCCGATGGGGCTCATACCCCGCCACT

CGAGCGACAGGCGTCTGCCCTCTTCCCACAAACCATTCCCTGGGCGCTCATCATCACGTGTCAACCAGTCACT

AACCACCTGTCAATAGGAAGCCGCCGAGCTCGGTAAGGGTTCCTTCAAGTACGCCTGGGTTCTTGACAAGCTC

AAAGCCGAGCGTGAGCGTGGTATCACCATTGATATCGCCCTCTGGAAGTTCGAGACTCCTCGCTACTATGTCA

CCGTCATTGGTATGTTGTCACCACTGCTGTCATCACATTCTCATACTAACATGGCTATCAGACGCTCCCGGTC

ACCGTGATTTCATCAAGAACATGATCACTGGTACTTCCCAGGCCGATTGCGCCATTCTCATCATTGCCGCCGG

EF1-R3

TACTGGTGAGTTCGAGGCTGGTATCTCCAAGGATGGCCAGACCCGTGAGCACGCTCTCCTTGCCTACACCCTT

GGTGTCAAGAACCTCATTGTTGCCATCAACAAGATGGACACCACCAAGTGGTCTGAGGCCCGTTACCAGGAGA

TCATCAAGGAGACCTCTTCTTTCATCAAGAAGGTCGGCTACAACCCCAAGGCTGTCGCTTTCGTCCCCATCTC

CGGTTTCAACGGTGAC

XXX : **Intron**

XXX : **Exon**

— — : Sequence present in Fusarium ID database (Fusarium MLST database)
