# Supplementary material for: Metabarcoding targeting the EF1 alpha region to assess Fusarium diversity on cereals
Source: PLoS One. 2019 Jan 11;14(1):e0207988. doi: 10.1371/journal.pone.0207988 (PMC6329491; doi:10.1371/journal.pone.0207988)
Supplement: S7 Fig — (PDF) [file pone.0207988.s007.pdf]

Pipeline according to Mothur MiSeq SOP

1) quality and trim of the reads for each file (3 files as example A, B, and C)

```
# fastq.info(fastq=uniq.seqs.A.fastq)
# fastq.info(fastq=uniq.seqs.B.fastq)
# fastq.info(fastq=uniq.seqs.C.fastq)
# trim.seqs(fasta=uniq.seqs.A.fasta, oligos=Forward.EF1.F2.oligos, qfile=uniq.seqs.A.qual, maxambig=0, maxhomop=6,  minlength=100, qwindowaverage=28, qwindowsize=25)
# trim.seqs(fasta=uniq.seqs.B.fasta, oligos=Forward.EF1.F2.oligos, qfile=uniq.seqs.B.qual, maxambig=0, maxhomop=6,  minlength=100, qwindowaverage=28, qwindowsize=25)
# trim.seqs(fasta=uniq.seqs.C.fasta, oligos=Forward.EF1.F2.oligos, qfile=uniq.seqs.C.qual, maxambig=0, maxhomop=6,  minlength=100, qwindowaverage=28, qwindowsize=25)
```

2) group the curated reads of the different files in one file

```
# make.group(fasta=uniq.seqs.A.trim.fasta-uniq.seqs.B.trim.fasta-uniq.seqs.C.trim.fasta, groups=a-b-c, output=merge.groups)
merge.files(input=uniq.seqs.A.trim.fasta-uniq.seqs.B.trim.fasta-uniq.seqs.C.trim.fasta, output=merge.ABCdef.fasta)
```

3) search and count unique sequences

```
# unique.seqs(fasta=merge.ABCdef.fasta)
# count.seqs(name=merge.ABCdef.names, group=merge.groups)
# summary.seqs(fasta=uniq.seqs.A.trim.fasta)
# summary.seqs(fasta=uniq.seqs.B.trim.fasta)
# summary.seqs(fasta=uniq.seqs.C.trim.fasta)
# summary.seqs(fasta=merge.ABCdef.fasta)
# summary.seqs(fasta=merge.ABCdef.unique.fasta)
# split.abund(fasta=merge.ABCdef.unique.fasta, name=merge.ABCdef.names, cutoff=1, accnos=true)
```

4) Search for chimera

```
# chimera.uchime(fasta=merge.ABCdef.unique.abund.fasta, name=merge.ABCdef.abund.names, reference=self)
# remove.seqs(accnos=merge.ABCdef.unique.abund.denovo.uchime.accnos, fasta=merge.ABCdef.unique.abund.fasta, name=merge.ABCdef.abund.names)
```

5) Classify against taxonomy EF1 reference file (**merge.reference.fasta; merge.taxonomy.fasta**)

```
# classify.seqs(fasta=merge.ABCdef.unique.abund.pick.fasta, template=merge.reference.fasta, taxonomy=merge.taxonomy.fasta, iters=1000, name=merge.groups, processors=12)
```

6) Distance matrix calculation

```
# pairwise.seqs(fasta=merge.ABCdef.unique.abund.pick.fasta, processors=12)
```

7) Clustering

cluster(column=merge.ABCdef.unique.abund.pick.dist, name=merge.ABCdef.abund.pick.names)

8) Create database

get.oturep(column=merge.ABCdef.unique.abund.pick.dist, name=merge.ABCdef.abund.pick.names, fasta=merge.ABCdef.unique.abund.pick.fasta, list=merge.ABCdef.unique.abund.pick.an.list, label=0.02)
classify.otu(taxonomy=merge.ABCdef.unique.abund.pick.taxonomy.wang.taxonomy, name=merge.ABCdef.abund.pick.names, list=merge.ABCdef.unique.abund.pick.an.list, reftaxonomy=merge.taxonomy.fasta)
create.database(list=merge.ABCdef.unique.abund.pick.an.list, label=0.02, repfasta=merge.ABCdef.unique.abund.pick.an.0.02.rep.fasta, repname=merge.ABCdef.unique.abund.pick.an.0.02.rep.names, constaxonomy=merge.ABCdef.unique.abund.pick.an.0.02.cons.taxonomy, group=merge.groups)

9) calculate error rate
merge.files(input=merge.ABCdef.Mock1.names-merge.ABCdef.Mock2.names-merge.ABCdef.Mock3.names-merge.ABCdef.Mock4.names-merge.ABCdef.Mock5.names, output=merge.names)
merge.files(input=merge.ABCdef.unique.abund.pick.Mock1.fasta-merge.ABCdef.unique.abund.pick.Mock2.fasta-merge.ABCdef.unique.abund.pick.Mock3.fasta-merge.ABCdef.unique.abund.pick.Mock4.fasta-merge.ABCdef.unique.abund.pick.Mock5.fasta, output=merge.fasta)
seq.error(fasta=merge.fasta, name=merge.names, reference=merge.reference.fasta, aligned=F)
